# Boosting Charge Storage in Ferri/Ferrocyanide‐Based Alkaline Redox Flow Batteries

**DOI:** 10.1002/anie.3588313

**Published:** 2026-09-22

**Authors:** Mahla Sarfaraz Khabbaz, Kuldeepsinh Raj, Lucas Zhang, Sepideh Biabanialitappeh, J. David Bazak, Nav Nidhi Rajput, Xiaoliang Wei

**Affiliations:** ^1^ School of Mechanical Engineering Purdue University Indianapolis Indiana USA; ^2^ Department of Materials Science and Chemical Engineering Stony Brook University Stony Brook New York USA; ^3^ Physical and Computational Sciences Directorate Pacific Northwest National Laboratory Richland Washington USA

**Keywords:** alkaline, ferri/ferrocyanide, lithium cation, polysulfide, redox flow battery, solubility

## Abstract

The ferri/ferrocyanide redox couple has long served as a benchmark catholyte in aqueous redox flow batteries (RBFs), particularly under highly alkaline conditions. However, the limited alkaline solubilities of commercially available Na^+^ and K^+^ forms constrain the attainable energy density of these systems. Here, we demonstrate that Li^+^ substitution substantially boosts the alkaline solubility of ferri/ferrocyanide, offering a promising route to overcome the long‐standing energy density limitation. Complementary spectroscopic and computational analyses reveal a distinct solubilization mechanism in which Li^+^ promotes mixed‐solvation and aggregate‐rich solution structures that sustain highly concentrated, fully dissolved electrolytes beyond the saturation behavior of Na^+^ and K^+^ analogs. When Li_3/4_Fe(CN)_6_ is paired with polysulfide or organic anolytes, the resulting flow cells demonstrate exceptional cycling stability and energy density in alkaline environments. Using symmetric flow cell configurations, we further elucidate the stability and decomposition mechanisms of the corresponding redox materials, revealing contrasting effects of electrolyte alkalinity: it compromises ferri/ferrocyanide stability while substantially enhancing polysulfide stability. These findings establish complementary molecular insights into redox materials solubilization and stabilization, providing a mechanistic foundation for developing energy‐dense and durable alkaline RBFs.

## Graphical abstract

By modifying the solvation environment, cation engineering through Li^+^ substitution of traditional K^+^ or Na^+^ boosts the alkaline solubility of the ferri/ferrocyanide catholyte redox couple, providing a viable pathway to overcome the long‐standing challenge of energy density limitations for aqueous redox flow batteries (RBFs).

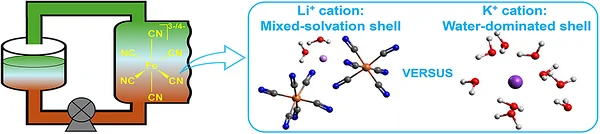

## 1 Introduction

Cost‐effective, large‐scale energy storage has been sought to ensure the reliability, resilience, and affordability of an increasingly dynamic power grid. Among the available technologies, redox flow batteries (RFBs) have attracted intensive attention because the stored power and energy can be scaled independently, offering exceptional flexibility in system design and ease of manufacturing. This feature makes RFBs particularly well suited for long‐duration energy storage (LDES) applications [1]. However, the widespread commercialization of RFBs has been hampered by practical techno‐economic challenges. The commonly studied all‐vanadium RFBs, while capable of long‐term stable operations, face a substantial cost barrier (currently estimated at $400–500 kWh^−1^), stemming primarily from the expensive vanadium oxide feedstock [2]. Organic redox‐active molecules constitute another major class of heavily investigated candidate materials, but their long‐term viability remains uncertain due to persistent challenges related to chemical instability, atmospheric sensitivity, and high synthetic cost [3, 4, 5].

To address these limitations, interest in traditional low‐cost inorganic redox materials, such as Fe, S, Zn, Br, Cr, and so forth, has been rejuvenated in recent years [6], among which the ferri/ferrocyanide redox couple (Fe(CN)_6_^3−/4−^) has emerged as one of the most intensively investigated catholytes [7]. This material has been paired with a wide variety of anolyte materials, including polysulfides [8], zinc [9], iron complexes [10], chromium chelates [11], and so forth, in pH‐neutral and alkaline aqueous RFBs. By cation exchange to an organic tetrabutylammonium, ferricyanide was even converted to an anolyte in nonaqueous RFBs due to the substantial negative shift of its redox potential in organic solvents [12]. In addition, other types of hexacyanometallates, such as Cr and Mn, have also been developed as redox materials or mediators for aqueous RFBs [13, 14, 15, 16, 17], suggesting the chemical robustness of such a structural skeleton. Compared to acidic or neutral electrolytes, alkaline media offer several advantages, such as a reduced risk of hydrogen evolution, good electrolyte conductivity, and moderate corrosion. However, catholytes having sufficient chemical stability in strongly alkaline electrolytes are scarce. Ferri/ferrocyanide is one of the few candidates that can be repeatedly cyclable under such conditions. Consequently, it has been frequently employed as a benchmark catholyte for evaluating newly developed organic anolytes, such as anthraquinone and phenazine, that performed well in highly alkaline environments [18, 19, 20]. A longstanding concern with ferri/ferrocyanide is whether it can withstand harsh alkalinity over long durations (months to years). Symmetric flow cells of K_3/4_Fe(CN)_6_ at pH 14 have shown capacity fade, which has been attributed to either CN^−^ displacement by OH^−^ in the coordination sphere or reduction of Fe(CN)_6_^3−^ by water with oxygen evolution [21, 22, 23, 24]. Although the exact degradation mechanism remains under debate, it has been demonstrated that using carbon electrodes with lower surface area can substantially mitigate capacity decay [21]. An even more severe drawback of commercially available ferri/ferrocyanide is the low maximum attainable concentrations, for example, < 0.8 M in pH‐neutral electrolytes and, more critically for alkaline RFBs, < 0.5 M at pH 14. The use of mixed Na^+^–K^+^ cations has increased these values to 1.6 and 1.5 M, respectively [25, 26]. We and the other teams have shown that Li^+^ possesses a strong ability to boost the solubilities of ferri/ferrocyanide in neutral electrolytes [27, 28, 29, 30]. Other cations, including NH_4_^+^, Mg^2+^, and Ca^2+^, exhibit similar effects but are chemically incompatible with high pH [29, 31]. As a result of these constraints, an excess of ferri/ferrocyanide in large electrolyte volumes was typically used to ensure it does not limit cell performance, which inevitably compromises the energy density of alkaline RFBs.

In this work, we elucidate the distinct solubilization and decomposition mechanisms governing Li^+^‐based alkaline RFBs, particularly those using ferri/ferrocyanide catholyte and polysulfide anolyte (denoted as S/Fe). Li_3/4_Fe(CN)_6_ exhibits exceptional solubility in alkaline electrolytes, reaching a maximum accessible concentration of 2.83 M at pH 14. Combined spectroscopic and computational analyses reveal that the Li^+^ cation induces unique mixed‐solvation and aggregate‐rich structures that differ markedly from those formed by Na^+^ and K^+^ cations, thereby enabling delayed saturation and unusually high ferri/ferrocyanide solubility. In parallel, systematic studies of symmetric Li_3/4_Fe(CN)_6_ and Li_2_S_2/4_ flow cells provide direct evidence for the degradation pathways of the two redox couples. Although Li_3/4_Fe(CN)_6_ exhibits limited chemical stability under strongly alkaline conditions, polysulfides are substantially more stable in alkaline media, accounting for the markedly extended cycling stability of alkaline S/Fe flow cells. These findings not only establish complementary mechanistic insights into redox materials solubilization and stabilization, but also demonstrate substantially higher redox concentrations and energy densities than Na^+^‐ and K^+^‐based analogs. Overall, this work provides a Li^+^‐enabled molecular and electrochemical framework for designing energy‐dense and durable alkaline RFBs.

## 2 Results and Discussion

### 2.1 Solubility and Solvation

The solubilities of anolyte and catholyte materials in both charged and discharged states govern the maximum accessible redox concentration and, consequently, the energy density of an RFB system. Following the Beer–Lambert law, ultraviolet–visible (UV–vis) spectroscopy was employed to measure the solubilities of the synthetic Li_3/4_Fe(CN)_6_ in 1 M LiOH (pH 14). As shown in Figure S1, the UV–vis absorption profiles of Li_3/4_Fe(CN)_6_ remain essentially unchanged as the electrolyte pH varies from neutral to strongly alkaline. The calibration curves relating UV–vis peak intensity to the concentration of Li_3_/_4_Fe(CN)_6_ in 1 M LiOH are presented in Figure S2. The peak intensities of Li_3/4_Fe(CN)_6_ samples that were diluted from saturation were interpolated in these calibration curves, yielding solubilities of 3.02 and 2.83 M, respectively. These solubility values are higher than those previously reported [27], possibly owing to variations in the completeness of cation substitution and the hydration states of raw materials, final products, and commercial LiOH. For comparison, the solubilities of commercial Na^+^ and K^+^ analogs in their corresponding 1 M hydroxide electrolytes were also measured; these results are summarized in Figure 1a. In general, ferrocyanide (Fe(CN)_6_^4−^) is less soluble than ferricyanide (Fe(CN)_6_^3−^) and dictates the maximum operation concentration in flow cells. In water, Na_4_Fe(CN)_6_, K_4_Fe(CN)_6_, (Na + K)_4_Fe(CN)_6_, and Li_4_Fe(CN)_6_ exhibit solubilities of 0.60, 0.68, 1.59, and 3.37 M, respectively. The presence of 1 M MOH (M = Li, Na, or K) decreases these solubilities as a result of the common ion effect; in pH 14 electrolytes, these values drop to 0.46, 0.36, 1.52, and 2.83 M, respectively. Impressively, for Li_4_Fe(CN)_6_, the solubility drop is insignificant, and the new solubility still translates into a high volumetric specific capacity of 75.8 Ah·L^−1^. Under both neutral and highly alkaline conditions, Li_4_Fe(CN)_6_ consistently exhibits solubilities at least five times those of the Na^+^ or K^+^ analogs, underscoring a unique advantage of Li^+^ counterions and enabling the realization of high‐energy‐density RFBs.

**FIGURE 1:**
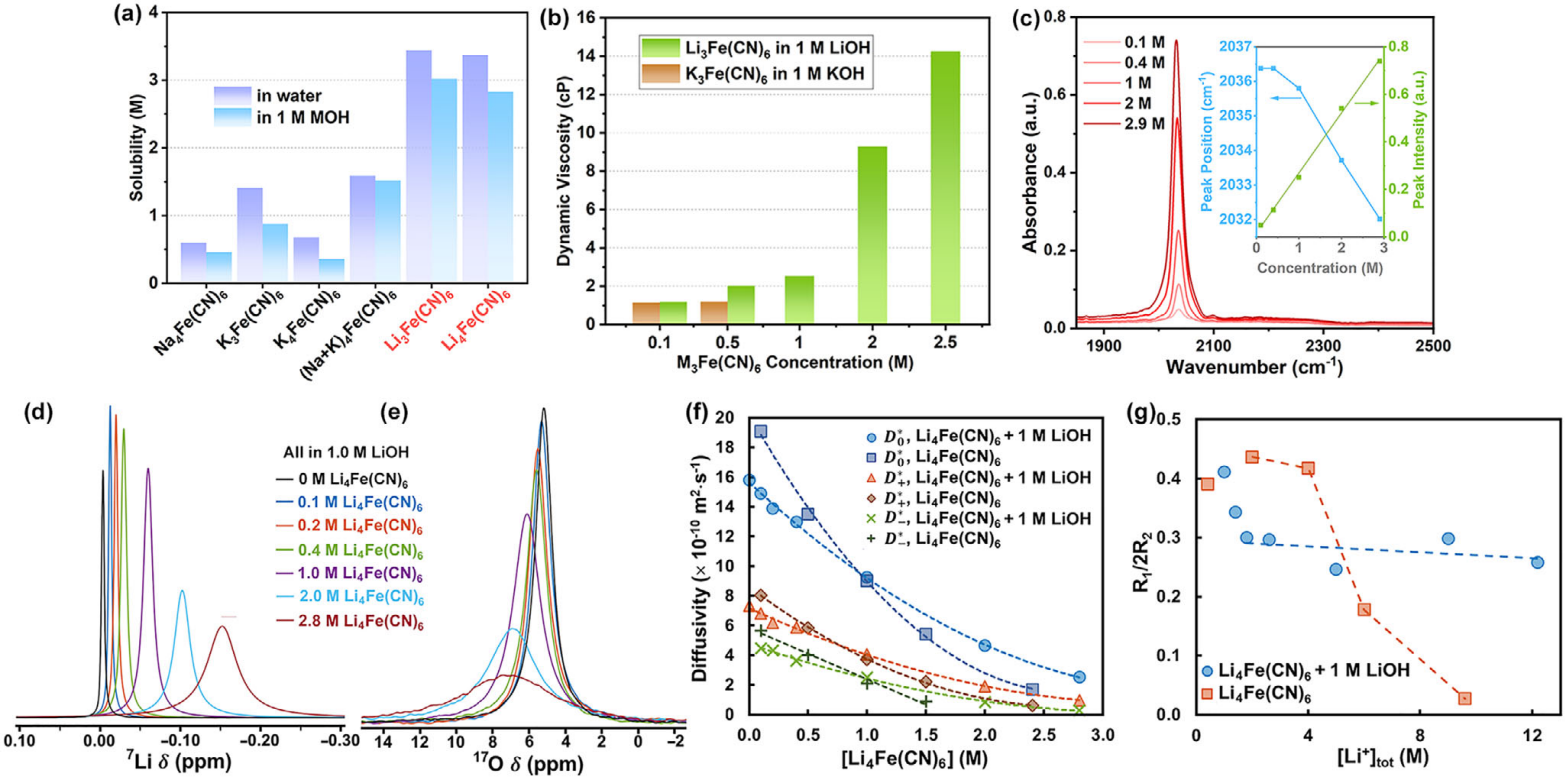
(a) Solubilities of M_3/4_Fe(CN)_6_ in water and in 1 M MOH (M = Li, Na, and K). (b) Dynamic viscosity as a function of M_3_Fe(CN)_6_ concentration in 1 M MOH (M = Li and K). (c) ATR‐FTIR spectra of different concentrations of Li_4_Fe(CN)_6_ in 1 M LiOH (inset: The *v*_CN_ peak position and intensity as a function of Li_4_Fe(CN)_6_ concentration). (d) ^7^Li NMR and (e) ^17^O NMR of a Li_4_Fe(CN)_6_ concentration series in 1 M LiOH. (f) Measured self‐diffusivities of Li^+^ ($D_{+}^{*}$), Fe(CN)_6_^4−^ ($D_{-}^{*}$), and H_2_O/OH^−^ ($D_{0}^{*}$) for the concentration series. (g) ^7^Li NMR relaxation ratios (*R*_1_ = 1/*T*_1_; *R*_2_ = 1/*T*_2_) of Li_4_Fe(CN)_6_ solutions (dashed lines are “guides to the eye”). Li_4_Fe(CN)_6_ diffusivities and relaxation ratios in water were reproduced from Ref. [30].

To assess mass transport characteristics of these electrolytes, the viscosities of a series of alkaline Li_3_Fe(CN)_6_ electrolytes in 1 M LiOH were experimentally measured (Figure 1b). The Li_4_Fe(CN)_6_ counterparts are expected to exhibit similar viscosity trends because of their closely related electrolyte compositions and solvation environments. The viscosity exhibits a strongly nonlinear dependence on Li_3_Fe(CN)_6_ concentration. As the Li_3_Fe(CN)_6_ concentration increases from 0.1 to 1 M, the viscosity gradually increases from 1.2 to 2.5 cP. Above 1 M, a pronounced increase is observed, reaching 9.3 cP at 2 M and 14.3 cP at 2.5 M. This strong deviation from linearity suggests that the intermolecular interactions and solution structure change substantially in the concentrated regime. Rather than behaving as independent, fully hydrated ions, Li^+^ and Fe(CN)_6_^3−^ progressively participate in cation–anion and ion–solvent associations. These collective interactions increasingly couple the motions of ionic species and solvent molecules, thereby restricting their effective translational mobility. Such structural characteristics become particularly marked at high redox concentrations, resulting in a rapid increase in viscosity and, consequently, greater resistance to mass transport. This behavior may become an important consideration for the rate capability of flow cells using concentrated Li_3_Fe(CN)_6_ electrolytes.

To elucidate the underlying role of the Li^+^ cation in boosting solubility, spectroscopic analyses, including attenuated total reflectance Fourier transform infrared (ATR‐FTIR) and nuclear magnetic resonance (NMR), were conducted to probe the solution chemistry of Li_4_Fe(CN)_6_ electrolytes. In general, the C≡N stretching vibration is highly sensitive to changes in solvating, H‐bonding, and electrostatic interactions [32, 33]. In the solution phase, stronger H‐bonding between water molecules and the terminal N atoms of Fe(CN)_6_^4−^ tightens the C≡N bonds, typically resulting in a blueshift of the C≡N stretching vibration (*ν*_CN_) [34, 35]. To minimize the interference from the intense absorption signal of water, the ATR mode was used to conduct FTIR measurements on alkaline Li_4_Fe(CN)_6_ electrolytes. As shown in Figures 1c and S3, Li_4_Fe(CN)_6_ exhibits a characteristic *ν*_CN_ at 2036.4 cm^−1^ [36], with the intensity increasing linearly with Li_4_Fe(CN)_6_ concentration. In the dilute concentration regime (0.1∼0.4 M), where water strongly interacts with Fe(CN)_6_^4−^ through CN^−^⋯H bonding [37], the position of the *ν*_CN_ band remains essentially unchanged, indicating a largely invariant solvation structure. As the Li_4_Fe(CN)_6_ concentration increases to 1 M and above, the *ν*_CN_ band undergoes a gradual redshift, suggesting progressive weakening of H‐bonding interactions. Concurrently, the CN^−^⋯Li^+^ ion association becomes increasingly significant, which reduces the extent of water‐mediated H‐bonding around Fe(CN)_6_^4−^. These ATR‐FTIR results indicate an evolving solvation environment in which the contribution of water decreases while Li^+^ coordination to Fe(CN)_6_^4−^ becomes increasingly pronounced at elevated concentrations.

NMR spectroscopy is a powerful analytical tool for probing solvation dynamics in liquid electrolytes [38]. Concentration‐dependent ^1^H, ^13^C, ^7^Li, and ^17^O NMR spectra of Li_4_Fe(CN)_6_ (Figures 1d,e and S4) provide insights into the solvation structures of Li^+^, Fe(CN)_6_^4–^, and H_2_O/OH^−^ species. At moderate OH^−^ concentrations such as 1 M LiOH, rapid proton exchange between H_2_O and OH^−^ results in a single exchange‐averaged ^17^O peak rather than separate signals for the two species [39]. NMR chemical shifts and linewidths reflect ensemble‐averaged local solvation environments, primarily determined by the immediate coordination sphere, with additional contributions from the outer solvation network. With increasing Li_4_Fe(CN)_6_ concentration, the ^1^H and ^17^O signals broaden and move to progressively higher or more deshielded chemical shifts, whereas the ^13^C and ^7^Li resonances are more shielded. The observed line broadening is consistent with increased viscosity and reduced molecular mobility in concentrated solutions, which is related to enhanced ion association and slower exchange dynamics. The systematic chemical shift variations suggest a restructuring of the local solvation environment, characterized by reduced water coordination and increased anion participation in the Li^+^ solvation shell. Compared with the corresponding ^7^Li NMR spectra obtained for Li_4_Fe(CN)_6_ in pure water [30], the extents of both line broadening and chemical shift variation in 1 M LiOH are smaller despite the higher overall Li^+^ concentration (Figure 1d). Such a spectral difference may suggest that OH^−^ competes with Fe(CN)_6_^4−^ and H_2_O for coordination within Li^+^ solvates. Notably, the changes in the Li_4_Fe(CN)_6_‐specific ^13^C and ^7^Li signals become pronounced only above approximately 0.4 M, indicating a threshold‐like onset of significant Li^+^–Fe(CN)_6_^4−^ association. These NMR trends are consistent with the ATR‐FTIR observation, collectively indicating a concentration‐mediated evolution of the electrolyte solvation structure.

To further elucidate how Li^+^ promotes the unusually high solubility of Li_3/4_Fe(CN)_6_, molecular dynamics (MD) simulations were performed on six electrolyte systems spanning relevant concentrations. To correlate well with NMR findings, we used Li_4_Fe(CN)_6_ electrolytes in computational investigations. Radial distribution function (RDF) analysis reveals a fundamental structural difference between the solvation environments of Li^+^ and K^+^ (Figures 2a–c and S5). In both neutral and alkaline electrolytes, the Li^+^–O(water) pair exhibits a sharp and well‐defined first‐shell peak at *r* ≈ 1.95–2.05 Å, consistent with the compact hydration structure expected for Li^+^ owing to its small ionic radius and high charge density [40]. The RDFs further show pronounced short‐range ordering between Li^+^ and both O(water) and N(CN^−^), indicating that Li^+^ interacts strongly with water while also forming direct contacts with ferrocyanide anions. Coordination number analysis reveals that Li^+^ maintains an approximately four‐coordinate first solvation shell across all investigated conditions (Figure 2d), but the identity of first‐shell neighbors evolves markedly with concentration. As Li_4_Fe(CN)_6_ approaches saturation, the Li–N(CN^–^) coordination increases while the Li–O(water) coordination correspondingly decreases, indicating progressive substitution of water by Fe(CN)_6_^4−^ within immediate Li^+^ proximity. These results suggest the formation of mixed‐solvation structures in which Li^+^ simultaneously coordinates water molecules and ferrocyanide anions, consistent with the pronounced nonlinear increase in viscosity observed in concentrated Li_4_Fe(CN)_6_ electrolytes (Figure 1b). In contrast, the K^+^–O (water) RDF exhibits a broader first‐shell peak at a substantially larger distance of r ≈ 2.7–2.8 Å, reflecting the weaker and more diffuse hydration characteristic of the larger K^+^ cation. K^+^ remains predominantly water‐coordinated with an average K^+^–O(H_2_O) coordination number near 7 and minimal direct association with Fe(CN)_6_^4−^, even at near‐saturation.

**FIGURE 2:**
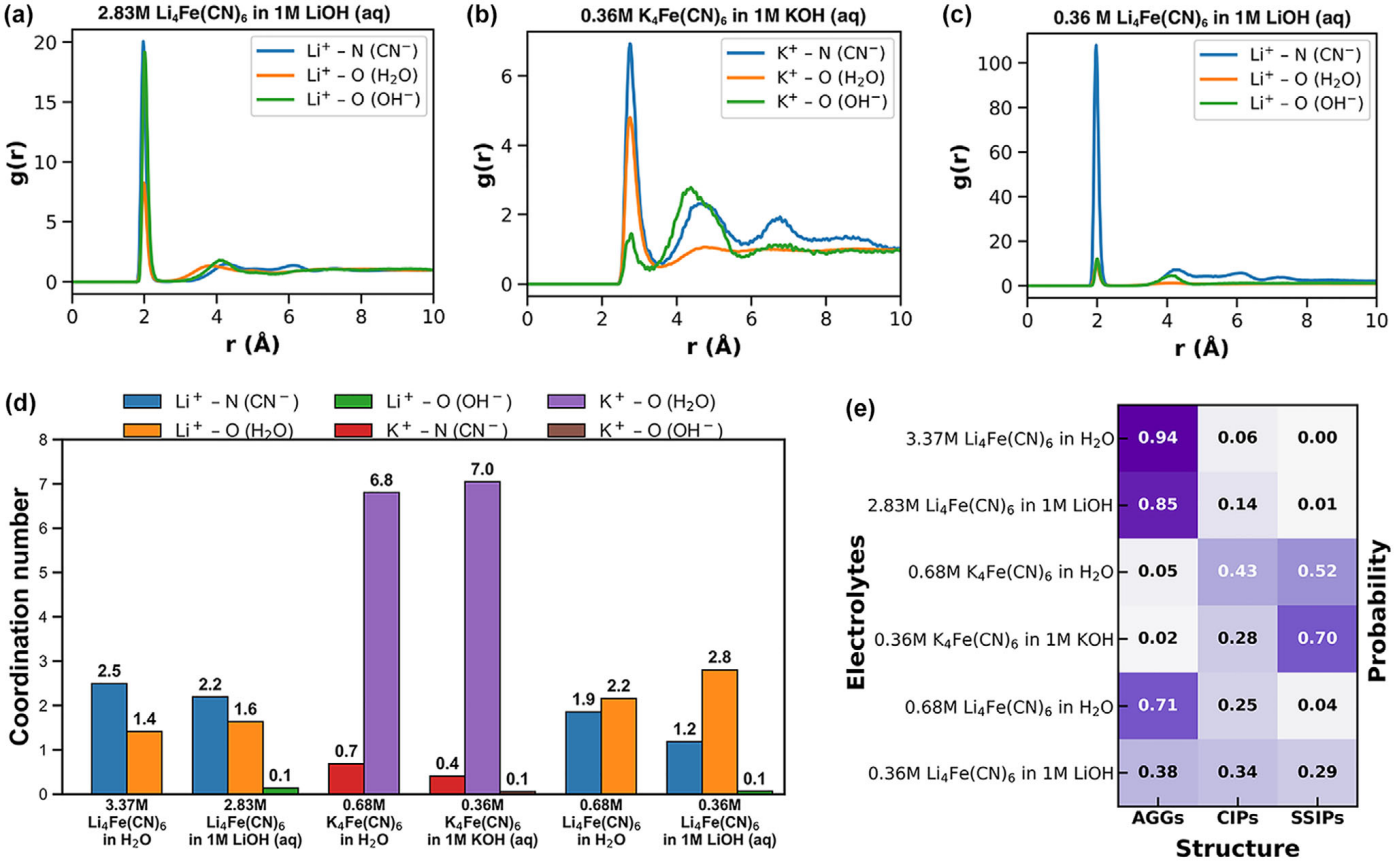
MD simulations of solution structures: (a–c) RDFs showing cation–O(water), cation–N(CN^−^), and cation–O(OH^−^) peaks in alkaline Li_4_Fe(CN)_6_ and K_4_Fe(CN)_6_ electrolytes. (d) First‐shell coordination numbers of Li^+^ and K^+^ with water, CN^−^, and OH^−^, respectively. (e) Speciation fractions of AGG, CIP, and SSIP in various Li_4_Fe(CN)_6_ and K_4_Fe(CN)_6_ electrolytes.

Speciation analysis further quantifies the nature of cation–anion associations. Ion speciation in solution‐phase electrolytes is commonly described in terms of three principal association motifs: solvent‐separated ion pairs (SSIPs), contact ion pairs (CIPs), and aggregate‐like ion clusters (AGGs) with an extended ion association network, which represent progressively stronger degrees of cation–anion association [41]. As shown in Figure 2e, in both neutral and alkaline Li_4_Fe(CN)_6_ electrolytes, AGGs dominate the solution‐state population, whereas the K_4_Fe(CN)_6_ electrolytes are primarily composed of CIPs and SSIPs with only limited AGG formation. This distinction indicates that Li^+^ promotes the formation of highly concentrated, aggregate‐rich yet fully dissolved structures [42, 43], suggesting a mechanism by which solution saturation is intrinsically delayed. Consistent with the speciation trends, cluster population analysis shows that the most probable local motifs in Li^+^‐based electrolytes contain multiple Fe(CN)_6_^4−^ anions, whereas the K^+^ counterparts are dominated by water‐rich motifs containing little or no Fe(CN)_6_^4−^ (Figure S6 and Table 1). For example, even in a dilute electrolyte of 0.36 M Li_4_Fe(CN)_6_ in 1 M LiOH, the most abundant cluster motif is [Li^+^][Fe(CN)_6_^4−^][H_2_O]_3_ (30.37%), and an additional 26.08% of clusters consist of [Li^+^][Fe(CN)_6_^4−^]_2_[H_2_O]_2_. These results indicate a mixed‐solvation environment in which Li^+^ bridges Fe(CN)_6_^4−^ while simultaneously retaining hydration water. Importantly, mixed‐solvation cluster motifs remain as the dominant configurations even in saturated Li_4_Fe(CN)_6_ electrolytes. The clusters [Li^+^][Fe(CN)_6_^4−^]_2_[H_2_O]_2_ (30.24%), [Li^+^][Fe(CN)_6_^4−^]_3_[H_2_O] (18.74%), and [Li^+^][Fe(CN)_6_^4−^]_2_[H_2_O]_3_ (11.00%) together account for nearly 60% of the total population. The persistence of mixed‐solvation motifs across a wide concentration range suggests that Li^+^ can organize compact, aggregate‐rich local structures while maintaining the electrolytes in a dissolved state. In contrast, K^+^ predominantly forms water‐coordinated environments with substantially weaker association to Fe(CN)_6_^4^^−^. Our viscosity measurement provides robust evidence to support this argument. At the same concentrations (0.1 and 0.5 M) in the respective 1 M supporting base, K_3_Fe(CN)_6_ exhibits lower viscosities than Li_3_Fe(CN)_6_ (Figure 1b), reflecting stronger Li^+^ interactions with counterions and water molecules. These trends are therefore consistent with the experimentally observed delayed saturation and exceptional solubility of Li_4_Fe(CN)_6_ electrolytes.

**TABLE 1:** Top three most probable clusters and OH^−^‐containing clusters in each electrolyte system with their corresponding population percentages.

Electrolyte	Cluster code	No. of Fe(CN)_6_^4−^	No. of water	Frequency (%)
3.37 M Li_4_Fe(CN)_6_ in H_2_O	1	2	2	28.50
	2	3	1	26.95
	3	3	2	10.16
2.83 M Li_4_Fe(CN)_6_ in 1 M LiOH	1	2	2	30.24
	2	3	1	18.74
	3	2	3	11.00
	OH^−^‐containing	—	—	12.65
0.68 M K_4_Fe(CN)_6_ in H_2_O	1	0	9	16.78
	2	1	8	13.13
	3	0	8	12.84
0.36 M K_4_Fe(CN) _6_ in 1 M KOH	1	0	9	19.62
	2	0	8	14.69
	3	0	10	13.98
	OH^−^‐containing	—	—	9.95
0.68 M Li_4_Fe(CN)_6_ in H_2_O	1	2	2	49.16
	2	1	3	23.8
	3	3	1	16.58
0.36 M Li_4_Fe(CN)_6_ in 1 M LiOH	1	1	3	30.37
	2	2	2	26.08
	3	0	4	21.38
	OH^−^‐containing	—	—	6.31

Cluster codes 1–3 are illustrated in Figure S6.

To further support these structural observations, density functional theory (DFT) binding energy calculations were performed for cation–water and cation–anion interactions. Because the isolated gas‐phase energy of a tetraanion such as Fe(CN)_6_^4−^ is not physically meaningful, the values reported here were obtained in implicit water using the SMD continuum solvation model; the corresponding gas‐phase values are tabulated in Table S2 for comparison. Li^+^ binds more strongly than K^+^ to both water and Fe(CN)_6_^4−^, thereby enabling simultaneous stabilization of hydration and cation–anion coordination environments. Specifically, the Li^+^–H_2_O interaction is 1.79× stronger than K^+^–H_2_O, while for Fe(CN)_6_^4−^, the difference in binding energy, ΔΔ*E* = *E*_bind(Li^+^) − *E*_bind(K^+^) = −41.0 kJ mol^−1^, indicating that Li^+^ binds Fe(CN)_6_^4−^ more favorably than K^+^ (Figure S7 and Table S2). Because forming a CIP in solution requires displacing a coordinating water, the relevant quantity is the exchange energy between the two coordination modes: Li^+^ favors anion over water coordination by 42.4 kJ mol^−1^ and K^+^ by 31.0 kJ mol^−1^, a differential of 11.4 kJ mol^−1^ (∼4.6 k_BT at 298 K). These energetic trends are consistent with MD analysis, supporting the argument that Li^+^ favors the mixed‐solvation structures.

Therefore, a weaker association does not, in itself, imply a higher solubility. Rather than by the extent of ion association in the dissolved state, solubility is governed by the balance between the lattice free energy of the solid and the hydration free energies of its constituent ions. Because Fe(CN)_6_^4−^ is a large polyatomic anion, the interionic separation in M_4_Fe(CN)_6_ is dominated by the anion, and the lattice energy would be expected to be comparatively insensitive to the cation, whereas cation hydration free energy scales inversely with ionic radius and differs steeply between Li^+^ and K^+^. Our DFT calculations (Figure S7) place the Li^+^–H_2_O interaction 1.79 times stronger than K^+^–H_2_O, a difference that would enter this balance four times over per formula unit. We therefore propose that the size mismatch between Li^+^ and Fe(CN)_6_^4−^ underlies the higher solubility of the Li^+^ salt, in line with the general observation that size‐mismatched salts are more soluble than size‐matched ones.

It is noteworthy that, in alkaline systems, OH^−^ appears in only a minority of first‐shell clusters (6.31%–12.65%), indicating that hydroxide is not a dominant inner‐shell coordinating species in either K^+^‐ or Li^+^‐based electrolytes (Figure 2d and Table 1). The presence of OH^−^ slightly reduces cation–water coordination, while LiOH and KOH primarily function as supporting electrolytes that establish the alkaline environment. This low occupancy reflects the limited availability of hydroxide rather than its exclusion from the coordination shell. In the saturated alkaline Li_4_Fe(CN)_6_ electrolyte, OH^−^ constitutes only 2.2% of all candidate first‐shell donor atoms, the water and hydroxide oxygens together with the six terminal nitrogens of each Fe(CN)_6_^4−^, and 1.8% in the dilute one. For a four‐coordinate Li^+^, random occupation of first‐shell sites in proportion to donor abundance would place at least one OH^−^ in the shell of 8.7% and 7.2% of cations, respectively, against observed values of 12.65% and 6.31%. The simulated populations are therefore of the same order as simple availability predicts; at 1 M, hydroxide is too dilute relative to water and ferrocyanide to become a significant first‐shell component, irrespective of its intrinsic charge density.

Taken together, these observations account for the delayed saturation of Li_4_Fe(CN)_6_ electrolytes. Saturation is reached when the free energy of an ion in solution approaches that of the same ion in the crystal, which conventionally occurs because rising salt content both exhausts the available solvent and progressively dehydrates the ions until their local environment resembles that of the solid. In the Li^+^ system, the second step is interrupted because Li^+^ binds water strongly and simultaneously binds Fe(CN)_6_^4−^ strongly; added salt is accommodated by converting solvent‐separated ions into hydrated bridging centers rather than by dehydrating them. Every dominant configuration at saturation, [Li^+^][Fe(CN)_6_^4−^]_2_[H_2_O]_2_, [Li^+^][Fe(CN)_6_^4−^]_3_[H_2_O] and [Li^+^][Fe(CN)_6_^4−^]_2_[H_2_O]_3_, retains first‐shell water. These soluble aggregates differ from the ion associations that precede precipitation in two respects. First, they are strongly anion‐rich, whereas precipitation requires locally stoichiometric, charge‐neutral assemblies containing four Li^+^ per Fe(CN)_6_^4–^; we propose that the resulting local charge imbalance disfavors coalescence into such nuclei. Second, they are structurally heterogeneous, spanning a broad distribution of distinct coordination environments rather than converging on a single dominant local structure. Consistent with this picture, the self‐diffusivities (*D*) of Li^+^, Fe(CN)_6_^4−^ and H_2_O/OH^−^, measured by NMR (Figure 1f), remain finite and vary systematically with concentration up to saturation, indicating that these aggregates remain mobile in solution. Aggregate‐dominated yet fully dissolved solution structures of this kind are characteristic of water‐in‐salt and related super‐concentrated electrolytes, in which strong cation‐anion association coexists with the liquid state [42, 43].

Despite only modest perturbation to the local solvation structures, NMR analysis shows that OH^−^ has a marked influence on the transport properties in Li_4_Fe(CN)_6_ electrolytes. As shown in Figure 1f, the self‐diffusivities (*D*) of Li^+^, Fe(CN)_6_^4−^, and solvent (H_2_O/OH^−^) species decrease continuously with Li_4_Fe(CN)_6_ concentration, reflecting the progressive increase in electrolyte viscosity. Notably, the decline in diffusivity is less severe in 1 M LiOH than in pure water, and the *D* value becomes consistently higher at Li_4_Fe(CN)_6_ concentrations above ∼1 M. Such behavior is advantageous for minimizing mass‐transport loss and improving the performance of concentrated RFB electrolytes. The enhanced transport properties are likely associated with OH^−^‐mediated modulation of the Li^+^ solvation environment, even though OH^−^‐containing solvates constitute only a minor fraction of the overall population (Table 1). Besides the above linewidth and chemical shift analysis, NMR relaxation measurements also provide evidence for the different ion association in alkaline electrolytes. For the quadrupolar ^7^Li nucleus, deviations of the *R*_1_/2*R*_2_ ratio from the extreme motional narrowing limit of 0.5 reflect a reduction in the symmetry of the local electric field gradient within the solvation shell and are commonly associated with increased ion association [44]. As clearly shown in Figure 1g, the *R*_1_/2*R*_2_ ratio decreases sharply with increasing Li_4_Fe(CN)_6_ concentration in pure water, whereas in 1 M LiOH it remains comparatively stable across the entire concentration range up to saturation. These observations indicate that alkaline conditions suppress the development of highly associated Li^+^ solvation environments. This interpretation is consistent with the MD‐derived speciation analysis (Figure 2e), which shows reduced AGG populations in alkaline electrolytes relative to their neutral counterparts. The resulting attenuation of extended ion‐association networks contributes to the enhanced diffusivity observed in alkaline Li_4_Fe(CN)_6_ solutions. Therefore, beyond the conventional advantages of mitigating hydrogen evolution and corrosion, alkaline media play a fundamental molecular‐level role in promoting favorable transport characteristics in concentrated Li_4_Fe(CN)_6_ electrolytes.

### 2.2 Electrochemical Performance

To evaluate the redox behavior in alkaline electrolytes and compare it with that under neutral conditions, cyclic voltammetry (CV) measurements were conducted using 10 mM Li_3_Fe(CN)_6_ in 1 M LiOH and 1 M LiCl, respectively. The corresponding results are shown in Figures 3a, S8, and S9. Also included are the CV curves of polysulfide that will be used as the anolyte in subsequent flow cells. As expected, the Fe(CN)_6_^3−/4−^ redox reaction is not proton‐coupled and is therefore independent of pH. Accordingly, Li_3_Fe(CN)_6_ exhibits a near‐identical redox potential of 0.32 V versus Ag/AgCl in both LiOH and LiCl electrolytes. By applying combined Randles–Sevcik and Nicolson analyses to the CV curves acquired at different scan rates [45], a diffusion coefficient (*D*) of 1.82 × 10^−6^ cm^2^·s^−1^ and a heterogeneous electron transfer rate constant (*k*_0_) of 1.41 × 10^−3^ cm·s^−1^ were obtained for Li_3_Fe(CN)_6_ in LiOH. These kinetic parameters are comparable to those measured in LiCl, namely a *D* of 3.15 × 10^−6^ cm^2^·s^−1^ and a *k*_0_ of 6.24 × 10^−3^ cm·s^−1^. This close similarity indicates that replacing a neutral supporting electrolyte with a strongly alkaline one does not significantly alter the intrinsic redox properties of Li_3_Fe(CN)_6_. Therefore, the rich prior studies of Fe(CN)_6_^3−/4−^ in neutral electrolytes [7] can be directly leveraged to understand the redox properties in alkaline environments.

**FIGURE 3:**
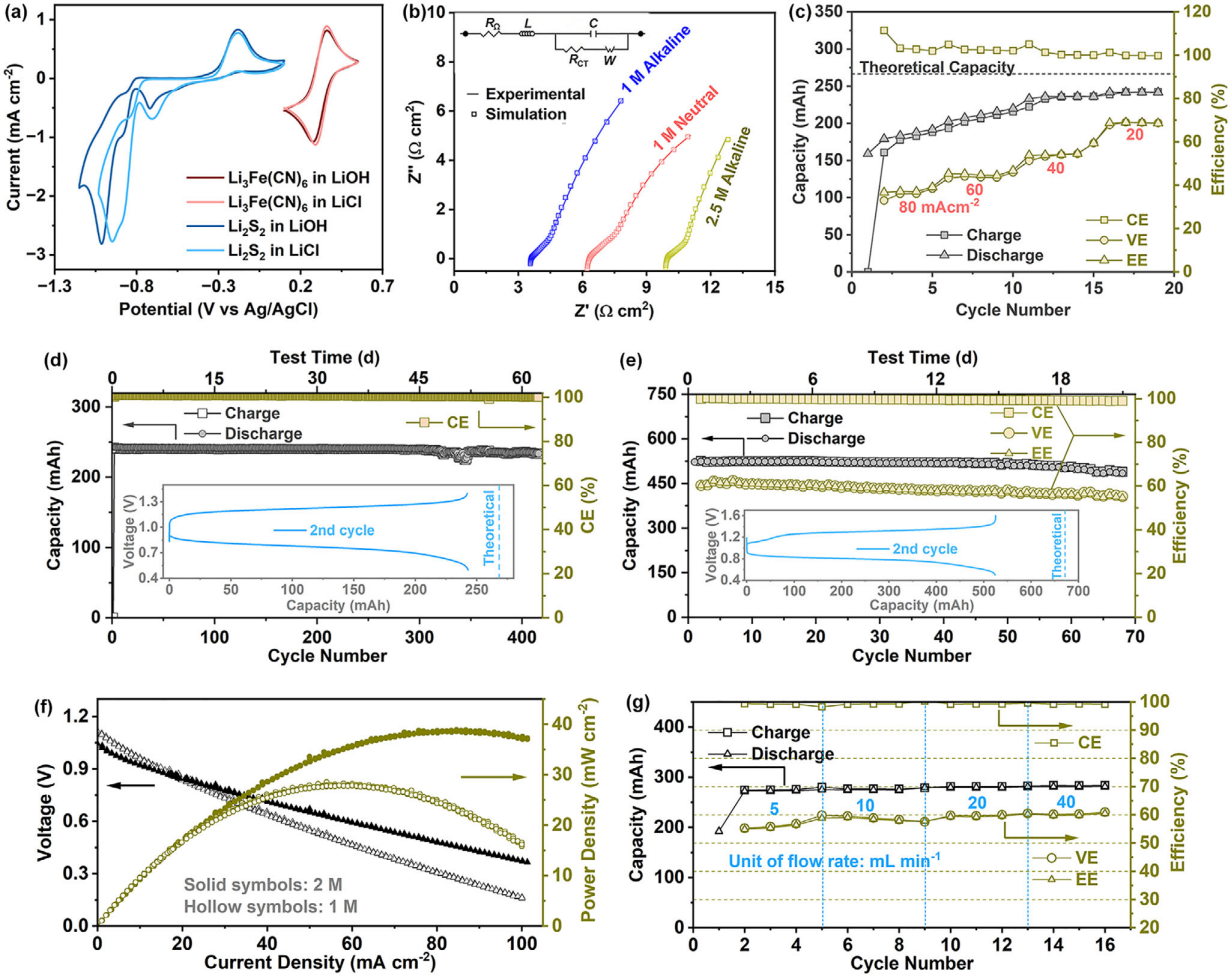
Electrochemical performance of the S/Fe system: (a) CV curves of 10 mM Li_3_Fe(CN)_6_ and 10 mM Li_2_S_2_ in 1 M LiOH and 1 M LiCl, respectively, both at a scan rate of 50 mV·s^−1^. (b) Experimental and simulated EIS spectra (Inset: equivalent circuit used for simulation with components: *R*_Ω_—ohmic resistivity; *R*_CT_—charge‐transfer resistivity; *L*—inductor; *C*—capacitor; *W*—Warburg impedance). (c) Rate performance and (d) cycling capacities and CE (at 20 mA·cm^−2^) of a 1 M S/Fe flow cell in 1 M LiOH. (e) Cycling capacities and efficiencies of a 2.5 M S/Fe flow cell in 1 M LiOH at 20 mA·cm^−2^. (f) Polarization curves and power densities of 1 and 2 M alkaline S/Fe flow cells at 20 mL·min^−1^ at 100% SOC. (g) Capacities and efficiencies of a 2 M alkaline S/Fe flow cell at 20 mA·cm^−2^ under different flow rates (theoretical capacity: 375 mAh).

To demonstrate the electrochemical performance in alkaline flow cells, Li_3/4_Fe(CN)_6_ catholyte was first paired with polysulfide anolyte (denoted as S/Fe). Sulfur originates mainly from fossil fuel processing and is extremely inexpensive; thus, its large‐scale use in the form of polysulfides in aqueous RFBs is being explored [46, 47, 48, 49]. Based on the Beer‒Lambert law with UV–vis spectroscopy (Figure S10), lithium polysulfides exhibit generally high solubilities in 1 M LiOH, that is, 9.0 M for Li_2_S, 7.38 M for Li_2_S_2_, and 3.56 M for Li_2_S_4_. As shown by the CV curves in Figure 3a, Li_2_S_2_ exhibits alike voltammetric waves in neutral and alkaline electrolytes, commonly with a major oxidation peak at −0.18 V versus Ag/AgCl and multiple reduction peaks. The strongly alkaline LiOH slightly shifts the major reduction peak from −0.95 V (in neutral LiCl) to −1.01 V versus Ag/AgCl. In addition, a couple of “shoulder” reduction peaks are present at −0.72 and −0.89 V versus Ag/AgCl, which are characteristic of coupled electrochemical‐chemical (EC) reaction mechanisms [50]. Assuming that the major redox peaks represent the S_2_^2−^ ↔ S_4_^2−^ + 2e^−^ reaction, a formal redox potential of −0.60 V versus Ag/AgCl was obtained, leading to a cell voltage of 0.92 V for the S/Fe system. The extremely wide peak separation (∼0.83 V) suggests slow redox kinetics, a long‐standing challenge for polysulfide conversion, which necessitates the use of electrocatalysts to expedite this redox reaction.

According to a prior report [21], electrodes having lower surface areas can mitigate capacity fading of symmetric K_3/4_Fe(CN)_6_ flow cells operated at pH 14 by reducing parasitic oxygen evolution reaction. Therefore, a commercial AvCarb HCB 1186 carbon fabric electrode (Figure S11) was employed in alkaline S/Fe flow cells; after thermal treatment, this electrode has a substantially lower surface area (0.61 m^2^·g^−1^) than the classical Sigracell GFD graphite felts (7.52 m^2^·g^−1^) [51]. To address the sluggish kinetics of polysulfide redox, heterogeneous metallic cobalt electrocatalyst‐decorated HCB 1186 electrodes (Co‐HCB) were chemically prepared and used at the anolyte side of S/Fe flow cells [8]. To reduce polysulfide crossover, a low‐ion‐exchange‐capacity Fumasep F1850 membrane, which exhibits a remarkable polysulfide retention capability [52], was assembled. Figure 3b plots the experimental and simulated Nyquist plots of S/Fe flow cells under various conditions, which were measured using electrochemical impedance spectroscopy (EIS). The alkaline S/Fe flow cell exhibits a lower area‐specific resistance (ASR), obtained by multiplying the high‐frequency resistance by the active cell area, than the neutral one (3.5 vs. 6.3 Ω·cm^2^), reflecting the higher electrolyte conductivity of the former. As shown in Figures 3c and S12, as the current density increases from 20 to 80 mA·cm–^2^, the coulombic efficiency (CE) remains nearly 100% while the voltage efficiency (VE) and energy efficiency (EE) drop from 69% to 37%; the catholyte material utilization also drops from 90% to 68% (anolyte used in excess). Impressively, the alkaline S/Fe flow cell demonstrates stable capacity for 416 cycles (corresponding to 63 days) with an average decay rate of 0.0092% per cycle or 0.061% per day (Figure 3d). In contrast, the capacity of a neutral S/Fe flow cell was maintained for only ∼100 cycles (∼17 days, Figure S13). The longer duration of stable cycling of the alkaline S/Fe flow cell suggests the drastic effects of electrolyte pH, reflecting the advantages of using alkaline electrolytes.

To achieve a good energy density, an alkaline S/Fe flow cell at a high redox concentration of 2.5 M in the presence of 1 M LiOH was tested at 20 mA·cm^−2,^ and the cycling result is plotted in Figure 3e. As shown in Figure 3b, a substantially higher ASR of 9.9 Ω·cm^2^ was obtained for this S/Fe flow cell, accompanied by a decreased catholyte material utilization ratio of 78%, compared to the 1 M flow cell. Based on the total electrolyte volume (i.e., 25 mL), a volumetric energy density of 27 Wh·L^−1^ was achieved for the charge process and that of 16 Wh·L^−1^ for discharge. These values are substantially lower than the theoretical value of 51 Wh·L^−1^, primarily because an excess polysulfide volume was used in this flow cell. Near‐100% CE was maintained throughout the test over 68 cycles that correspond to 21 days. Relatively stable capacity was retained during the cycling, but a fluctuating capacity drop followed. The VE and EE continuously decreased from the initial 62% to 55% at the end of cycling. For both the 1 and 2.5 M S/Fe flow cells, gradual increases in the overpotentials were observed as the cycling proceeded (Figure S14), which explains the VE drops and indicates increased ASRs in both cases.

The high ASR of this 2.5 M S/Fe flow cell is consistent with the markedly higher viscosity and associated mass‐transport resistance of the concentrated alkaline Li_3_Fe(CN)_6_ electrolyte (Figure 1b,f), which can adversely affect RFB performance. The measured peak power density of a 2 M alkaline S/Fe flow cell at 100% state of charge (SOC) is 28.2 mW·cm^−2^, substantially lower than the 38.8 mW·cm^−2^ achieved with the 1 M flow cell (Figure 3f). The 2 M flow cell also demonstrates an inferior rate capability (Figure S15). As the current density increases from 20 to 60 mA·cm^−2^, the EE decreases from 60% to 25%, and the catholyte utilization decreases from 74% to 68% (with widened voltage cutoffs). These performance losses highlight the severe cell polarization arising from the substantial mass‐transport limitation of the highly viscous electrolyte. The high viscosity can also increase the hydraulic resistance and pumping energy consumption, introducing an additional energy penalty. Previous studies of kW‐scale acidic vanadium RFB stacks, which typically employ electrolytes with viscosities below 8 cP [53], have demonstrated that electrolyte pumping can account for up to 10% of system power losses [54, 55]. The higher viscosity of concentrated Li_3_Fe(CN)_6_ electrolytes is therefore expected to further increase the energy losses associated with electrolyte pumping. Thus, viscosity represents an important practical consideration when evaluating the attainable energy and power densities of high‐concentration Li_3/4_Fe(CN)_6_ RFBs.

To balance performance gains and pump loss under concentrated conditions, the cycling performance of a 2 M alkaline S/Fe flow cell was evaluated at 20 mA·cm^−2^ over a range of flow rates (Figure 3g). As the flow rate increases from 5 to 20 mL·min^−1^, the voltage curves (Figure S16) exhibit progressively reduced overpotential, accompanied by gradual increases in the average EE from 55.4% to 59.4%. Above 20 mL·min^−1^, the cell efficiency remains essentially unchanged, with the voltage curves nearly completely overlapping. According to prior reports [56, 57], under laminar flow conditions, the pressure drop is approximately proportional to flow rate, resulting in a near‐quadratic dependence of pumping power on flow rate. Thus, given the marginal efficiency gains at higher flow rates, operating at the lower end of the tested range is advantageous for minimizing pump loss in highly concentrated S/Fe flow cells.

These results highlight an inherent trade‐off between electrolyte concentration and cell efficiency. While concentrated electrolytes are attractive for maximizing energy density, their increased viscosity can exacerbate polarization and hydraulic losses, potentially offsetting part of the benefit of using high redox concentrations. Consequently, moderating the electrolyte concentration could provide a favorable balance between charge capacity and overall battery performance under practical operating conditions.

To assess the broader applicability of Li_3/4_Fe(CN)_6_, we paired it with an organic anolyte and investigated the resulting flow cells in an alkaline environment. Specifically, 7,8‐dihydroxyphenazine‐2‐sulfonic acid (DHPS), a phenazine‐based anolyte molecule having a two‐electron redox activity, was chosen for this purpose [19]. In 1 M LiOH, DHPS exhibits a solubility of 1.58 M, corresponding to an electron concentration of 3.16 M and an accessible volumetric capacity of 84.7 Ah·L^−1^. As shown in Figure 4a, DHPS displays a redox potential as low as −1.06 V versus Ag/AgCl, resulting in a full‐cell voltage of 1.38 V when paired with ferrocyanide catholyte (denoted as DHPS/Fe). Based on these parameters, a theoretical volumetric energy density of 55 Wh·L^−1^ can be achieved at the respective solubility limits. In previous studies of new organic anolyte molecules that favor alkaline conditions, K_4_Fe(CN)_6_ has commonly served as the catholyte, but its limited solubility often requires the use of large volumes [7]. In contrast, the high alkaline solubility of Li_4_Fe(CN)_6_ provides a major advantage, potentially enabling high attainable energy density.

**FIGURE 4:**
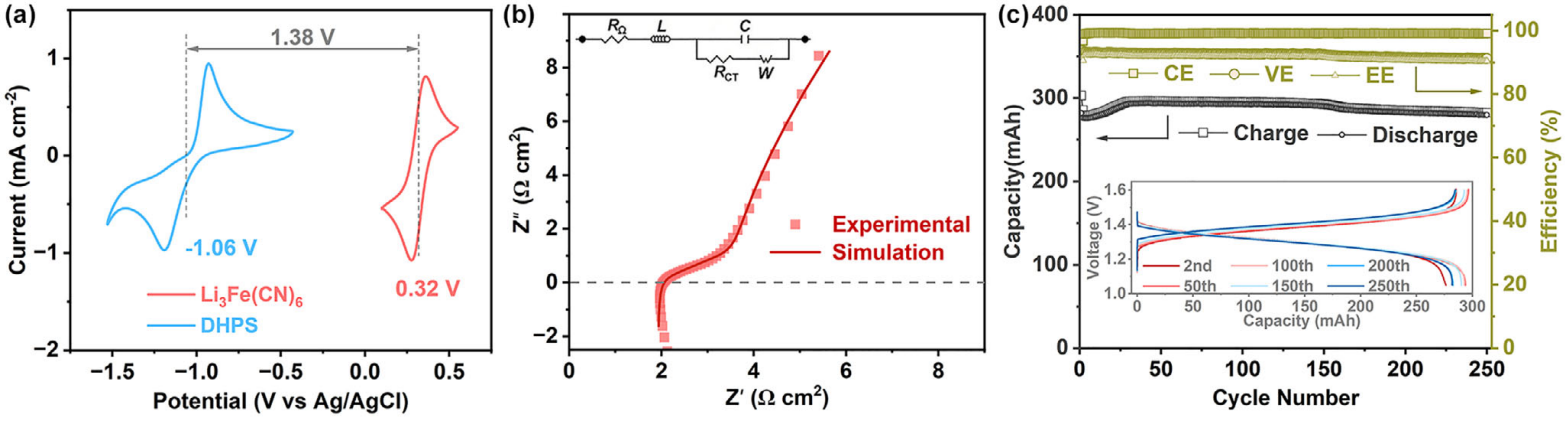
Electrochemical performance of the DHPS/Fe system: (a) CV curves of 10 mM Li_3_Fe(CN)_6_ and 5 mM DHPS, respectively, in 1 M LiOH at 50 mV·s^−1^. (b) Experimental and simulated EIS spectra (Inset: equivalent circuit). (c) Cycling capacities and efficiencies (inset: voltage curves) of a DHPS/Fe flow cell using 1 M DHPS anolyte and 1 M Li_4_Fe(CN)_6_ in 1 M LiOH at 30 mA·cm^−2^.

Both DHPS and ferrocyanide possess highly anionic structures, which are anticipated to induce strong Donnan exclusion effects and thereby effectively suppress through‐membrane crossover [58]. Taking advantage of this feature, the more ion‐conductive Nafion 212 membrane was assembled in the DHPS/Fe flow cell. Because of the potential tautomerization‐based decomposition of charged phenazine compounds [59], 1 M DHPS anolyte (in excess) and 1 M Li_4_Fe(CN)_6_ catholyte were used, making the catholyte the performance‐limiting side. As shown in Figure 4b, a lower ASR of 2.0 Ω·cm^2^ was extracted from the Nyquist plot, indicating favorable transport characteristics. Figure 4c presents the cycling performance of this flow cell tested at a constant current density of 30 mA·cm^−2^ for 250 cycles, corresponding to 31 days of continuous operation. This flow cell delivered an average CE of 99.0% together with a high average VE of 91.3%, reflecting both minimal crossover loss and low cell resistance. This result serves as a rigorous indication of the primary advantage conferred by the high solubilities of ferrocyanide catholyte in high pH electrolytes. Moreover, the capacity was well maintained throughout the cycling, with an average fade rate of 0.015% per cycle or 0.124% per day (calculated using the highest capacity, at the 32nd cycle, as the initial capacity). Such stable cycling highlights the excellent chemical robustness of both redox materials in strongly alkaline media, as well as their favorable anti‐crossover characteristics.

The cycling performance of alkaline S/Fe and DHPS/Fe flow cells reveals the pronounced advantages offered by Li^+^ cation in ferri/ferrocyanide‐based RFBs. The cation substitution preserves the intrinsic redox attributes of redox materials and, more importantly, the boosted solubilities enable alkaline RFBs to better demonstrate their performance by simultaneously accessing higher energy density and better efficiency. It is noted that the alkaline environment also leads to improved cycling stability relative to neutral conditions, particularly for the S/Fe system. The underlying mechanisms responsible for the extended stability are discussed in the next section. Moreover, alkaline RFBs will incur an additional cost associated with the use of LiOH (see an approximate cost analysis for S/Fe systems in Section S3 and Table S3). Despite this added cost, the overall material cost per unit of stored energy ($43.8 kWh^−^^1^) remains substantially lower than that of vanadium RFBs ($212 kWh^−^^1^) [60], demonstrating the promising techno‐economic potential of S/Fe RFBs.

### 2.3 Stability Analysis

Capacity fade in flow cells generally arises from two primary factors: (i) chemical decomposition and (ii) irreversible through‐membrane crossover of redox materials. Here, the S/Fe system was used as a model platform to systematically diagnose the degradation pathways and elucidate the dominant factors that govern cycling stability. We will investigate the Li_3/4_Fe(CN)_6_ catholyte and the Li_2_S_2/4_ anolyte separately to decouple their effects.

#### 2.3.1 Chemical Stability of Li_3/4_Fe(CN)_6_

Motivated by the prior studies on K_3/4_Fe(CN)_6_ in alkaline environments [21, 22, 23, 24], the chemical stability of Li_3/4_Fe(CN)_6_ under similar conditions warrants careful investigation. Symmetric flow cells, owing to their ability to minimize irreversible crossover, have been widely employed to probe the chemical stability of redox materials during RFB operation [61, 62]. We have previously used this method to confirm the exceptional stability of Li_3/4_Fe(CN)_6_ under pH‐neutral conditions [30], and here extend it to characterize it under alkaline environments. A symmetric Li_3/4_Fe(CN)_6_ flow cell was assembled with a 2 M total redox concentration in 1 M LiOH on both sides and a Li^+^‐form Nafion 212 membrane. The cell was repeatedly cycled at a current density of 20 mA·cm^−2^ to assess long‐term durability. As shown in Figure 5a, the cycling of this alkaline cell exhibits a continuous capacity fade with an average rate of 0.096% per cycle or 0.658% per day. The voltage curves along the cycling indicate an increase in cell overpotential (Figure S17). In stark contrast, a neutral symmetric flow cell operated under otherwise identical conditions displays near‐constant capacities through 500 cycles (i.e., ∼89 days). The test result agrees well with the prior reports of the pH‐induced capacity trends in symmetric K_3/4_Fe(CN)_6_ flow cells [21, 22, 23, 24]. Therefore, the alkaline instability of Li_3/4_Fe(CN)_6_ is anticipated to be a nonnegligible factor responsible for the limited cycle life of S/Fe flow cells, especially with concentrated redox electrolytes.

**FIGURE 5:**
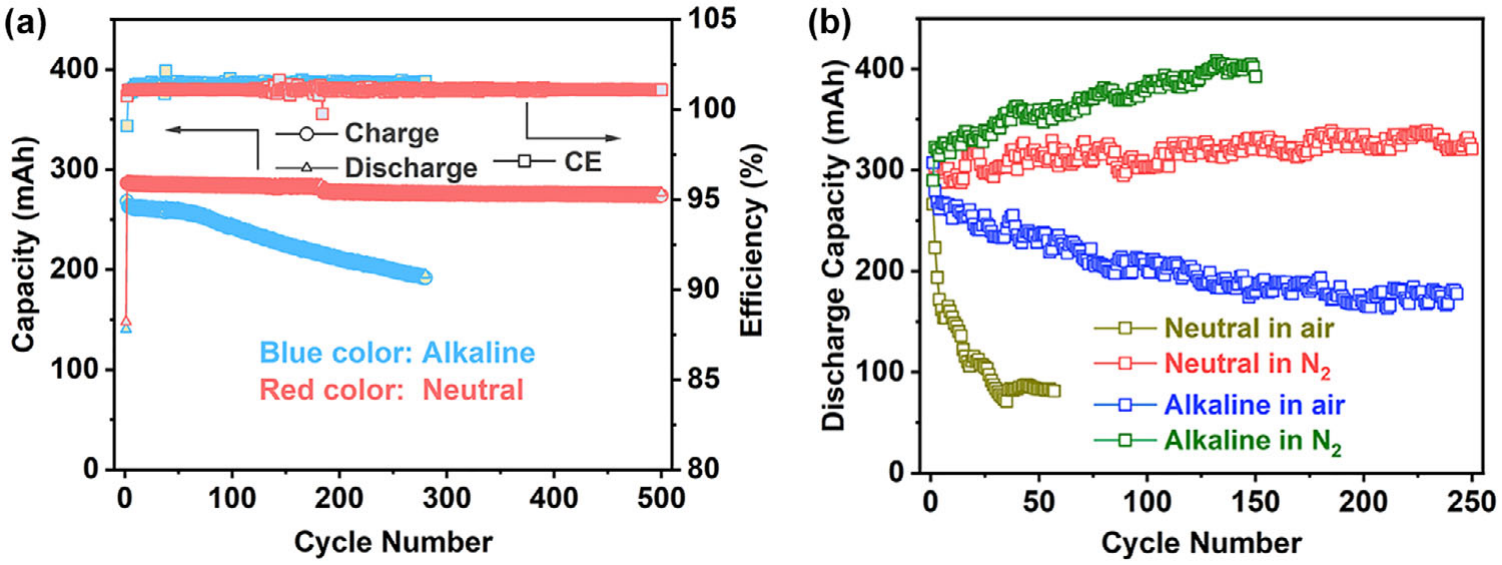
Cycling tests of symmetric flow cells: (a) Capacity and CE of 2 M alkaline and neutral symmetric Li_3/4_Fe(CN)_6_ cells at 20 mA·cm^−2^ (The minor capacity drop at the 184th cycle was due to replacement of pump tubing to avoid over‐abrasion); (b) Discharge capacity of alkaline and neutral symmetric Li_2_S_2/4_ flow cells at 20 mA·cm^−2^ in air and N_2_ atmospheres, respectively. The flow cells used 1 M Li_2_S_2_ (10 mL) on one side and 0.5 M Li_2_S_4_ (10 mL) on the other side.

#### 2.3.2 Chemical Stability of Polysulfides

As a well‐recognized decomposition route, polysulfides are thermodynamically susceptible to hydrolysis, which can generate HS^−^ and, under appropriate conditions, gaseous H_2_S [63, 64, 65]. Operation in strongly alkaline media is expected to suppress hydrolysis‐driven polysulfide decomposition. Moreover, polysulfide electrolytes are governed by complex disproportionation/comproportionation equilibria, resulting in the simultaneous presence of multiple S_n_^2−^ species (*n* = 1∼5) [66]. Longer‐chain polysulfides (*n* ≥ 6) are prone to aggregate into colloidal sulfur and ultimately elemental sulfur precipitation, causing irreversible polysulfide loss [67]. After cycling of a 1 M neutral S/Fe flow cell, field‐emission scanning electron microscopy (FESEM) was used to characterize the cycled electrodes. As shown in Figure S18, solid deposits were observed on the anolyte‐side HCB electrode, and energy‐dispersive x‐ray spectroscopy (EDX) confirmed that these solids were predominantly sulfur. This observation is consistent with our previous cycling test of a 2 M neutral S/Fe flow cell using different GFD graphite felt electrodes [30], in which thick layers of sulfur deposition were observed on the anolyte electrode after cycling (Figure S19). These results identify elemental sulfur as a major side product associated with polysulfide decomposition. The markedly extended cycling stability of alkaline S/Fe flow cells suggests that polysulfides exhibit greater chemical stability in alkaline electrolytes, consistent with prior reports on alkaline polysulfide‐based RFBs [26, 68, 69, 70]. To gain further insights, symmetric polysulfide flow cells were evaluated under neutral and alkaline conditions to compare their cycling stabilities.

The symmetric Li_2_S_2/4_ flow cells were assembled with 1 M Li_2_S_2_ (10 mL) on one side and 0.5 M Li_2_S_4_ (10 mL) on the other side, assuming a nominal half‐cell reaction: 2 Li_2_S_2_ ↔ Li_2_S_4_ + 2 Li^+^ + 2 e^−^. A constant current density of 20 mA·cm–^2^ was used to cycle these cells. The test data are plotted in Figures 5b and S20. First, the symmetric flow cells provide deeper insights into the redox behavior of polysulfides. Despite a theoretical capacity of 268 mAh based on the loaded polysulfide electrolytes, higher initial capacities are commonly observed in all these symmetric flow cells; those tested in an inert N_2_ atmosphere access even higher capacities during cycling. This result suggests that polysulfide redox may proceed to a broader speciation range, possibly involving S^2−^ and S_5_^2−^ species. Second, the cell atmosphere demonstrates a decisive effect on cycling stability. Compared to the rapid capacity fade in air, the N_2_ environment produces stable cycling, even with capacity increase, regardless of electrolyte pH. This finding strongly suggests that oxidation by leaked air/oxygen is a pivotal stability‐determining factor for polysulfides. In polysulfide‐based RFBs, this adverse effect is often obscured by the use of excessive polysulfide anolyte and thus has been underestimated [47]. Air oxidation of polysulfides is complex; the identity of the product depends on the S:O_2_ ratio and follows a trend from elemental S to S_2_O_3_^2−^, SO_3_^2−^, and finally SO_4_^2–^ as the O_2_ supply increases [71, 72, 73]. Under RFB conditions, concentrated polysulfides are mechanically sealed with slowly leaked air, resulting in ultrahigh S:O_2_ ratios and O_2_‐starved environments. Thus, we anticipate that partial oxidation is prominent with elemental S as the dominant product. This argument agrees well with the observation of sulfur deposits on the anolyte‐side electrodes after cycling of S/Fe flow cells (Figure S19) [30].

Third, when operated in air, the alkaline symmetric cell exhibits a significantly slower capacity fade than the neutral one. The former has an average fade rate of 0.162% per cycle or 1.264% per day, versus that of 1.219% per cycle or 17.879% per day for the latter. The better alkaline stability of polysulfides is ascribed to a combination of thermodynamic and kinetic factors. In a strongly alkaline environment (pH 14), the hydrolysis reaction is alleviated. The oxidizing power of O_2_ is weaker due to negative shifting of its redox potential, lack of proton‐coupled redox pathways, and milder reactive intermediates (O_2_^•−^) [74]. The “nascent” sulfur formed from air oxidation can partially be converted back to polysulfides, potentially via the routes shown in Equations (1) and (2) [75, 76]. The sulfur redissolution could retard polysulfide loss, curtail overpotential rise, and extend cycle life of S/Fe flow cells.

(1) $$4S+4{\text{OH}}^{-}\to S_{2}O_{3}^{2-}+2{\text{HS}}^{-}+H_{2}O$$

(2) $$\left(n-1\right)S+\text{HS}\text{--}+{\text{OH}}^{-}\to S_{n}^{2-}+H_{2}O$$

#### 2.3.3 Crossover of Polysulfides

Irreversible crossover of polysulfide species through the membrane into the catholyte compartment represents one of the most critical challenges for polysulfide flow cells, particularly when concentrated electrolytes are used [47, 48, 49]. The permeated polysulfides can react with the oxidized catholyte to produce elemental sulfur precipitates, increasing cell resistance, causing material loss and capacity fade, and potentially clogging flow channels. To mitigate this issue, a variety of advanced membranes have been developed [70, 77, 78]. We previously demonstrated that the F1850 membrane, featuring narrow ion channels, exhibits a strong ability to suppress polysulfide crossover under neutral conditions [52].

Here, we examine whether an alkaline environment influences polysulfide crossover through this membrane using an H‐shaped permeation cell (Figure 6a). The crossover flux was quantified through UV–vis characterizations of the permeate electrolyte collected at designated time intervals. The concentration of permeated Li_2_S_2_ (*C*_t_) was determined by interpolating the measured UV‐vis absorbance into the calibration curve shown in Figure S21. The through‐membrane permeability (*P*) of Li_2_S_2_ is calculated from the slope of the linear fit of Ln(1 − *C*_t_/*C*_0_) versus time (*t*) [70], as illustrated in Figure 6b,c. The measured *P* in a pH 14 environment is 4.51 × 10^−6^ cm^2^·d^−1^, which is moderately lower than that obtained under neutral conditions (8.19 × 10^−6^ cm^2^·d^−1^). This reduction may arise from OH^−^ anions diffusing into the membrane and generating additional Donnan exclusion effects that hinder polysulfide permeation [79]. However, the improvement is relatively modest. Considerable polysulfide crossover was still observed in alkaline S/Fe flow cells, as indicated by the formation of yellow precipitates in the catholyte compartment after cycling (Figure S22). EDX analysis confirmed that the identity of these solid deposits was predominantly elemental sulfur (Figure S23). Li_3_Fe(CN)_6_ exhibits negligible crossover through F1850 in neutral electrolyte [30], and similar behavior is expected under alkaline conditions due to the highly charged polyanionic nature of Fe(CN)_6_^3−^.

**FIGURE 6:**
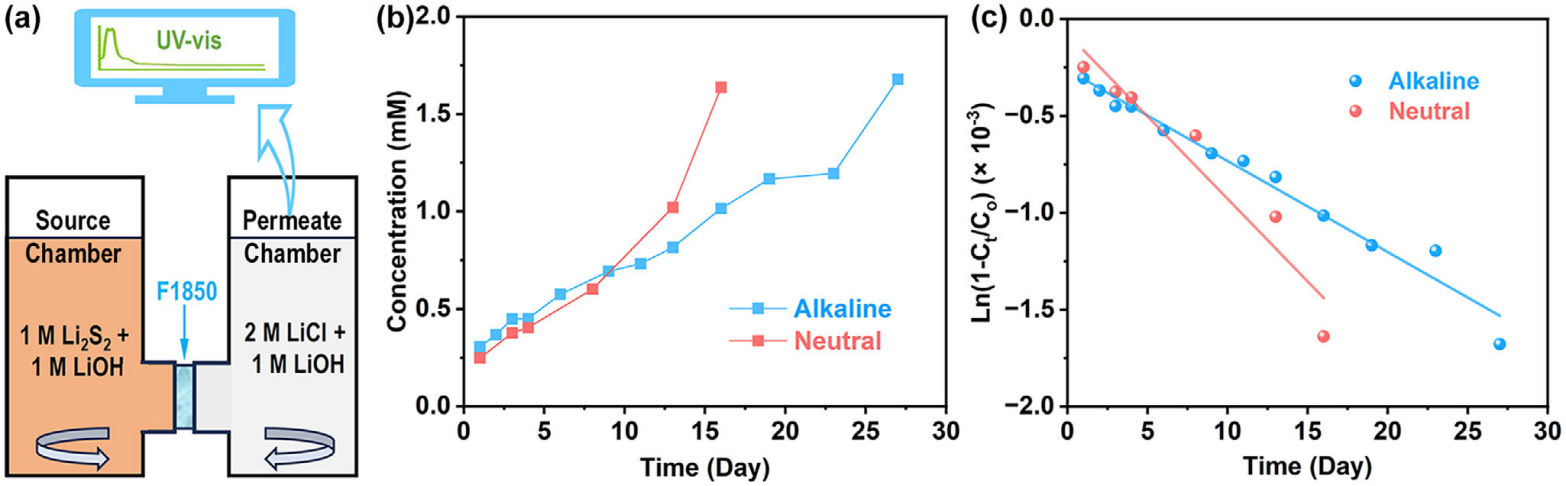
Li_2_S_2_ crossover test: (a) Schematic illustration of the permeation cell. (b) The permeated Li_2_S_2_ concentrations and (c) the derived Ln(1 − *C*_t_/*C*_0_) as a function of time in both neutral and alkaline environments.

Please note that the passive H‐cell permeability test primarily measures the concentration gradient‐driven diffusion of polysulfides through the membrane and does not capture the electric field‐driven electromigration and electroosmotic transport. The latter two mechanisms contribute during RFB operation and generally increase with current density [80, 81]. Therefore, the actual polysulfide crossover under operating conditions is expected to exceed the H‐cell permeation, particularly at high current densities. Although the H‐cell test cannot quantify the total crossover in operating flow cells, it provides a useful and consistent basis for comparing the intrinsic polysulfide permeability of the membrane under neutral and alkaline conditions.

## 3 Conclusion

In summary, we have demonstrated that cation engineering with Li^+^ substitution effectively boosts the solubility of the ferri/ferrocyanide redox couple in alkaline electrolytes, thereby overcoming a key energy density bottleneck for aqueous RFBs that conventionally employ Na^+^/K^+^ forms of this catholyte. Complementary NMR, ATR‐FTIR, MD simulations, and DFT calculations reveal a distinct Li^+–^modified solvation environment underlying this solubility enhancement. Compared with K^+^, Li^+^ interacts more strongly with water molecules and the Fe(CN)_6_^4−^ anion, stabilizing both hydration and cation–anion association and generating mixed‐solvation structures across a broad concentration range. In contrast to the water‐dominated solvation shell observed for K^+^ analogs, this unique solvation behavior enables Li^+^ electrolytes to sustain aggregate‐rich yet fully dissolved solution structures even at very high Li_4_Fe(CN)_6_ concentrations, thereby delaying saturation. The resulting exceptional alkaline solubility of Li_3/4_Fe(CN)_6_ provides a practical pathway toward accessing competitive energy densities in RFB systems that employ high‐capacity polysulfide or organic anolytes.

Switching from neutral to alkaline conditions markedly improves the cycling stability of Li^+^‐based S/Fe flow cells. Symmetric flow cell tests further reveal that this enhanced capacity retention is primarily associated with the greater chemical stability of polysulfides in alkaline media, which more than compensates for the inferior caustic durability of Li_4_Fe(CN)_6_. In particular, partial oxidation induced by air leakage is identified to be an important polysulfide decomposition pathway, resulting in the formation of elemental sulfur deposits. This oxidative degradation is substantially suppressed under alkaline conditions, providing a mechanistic explanation for the markedly extended cycle life of alkaline S/Fe flow cells.

## Author Contributions

**Mahla Sarfaraz Khabbaz**: methodology, data curation, investigation, validation, formal analysis, visualization, writing – review and editing. **Kuldeepsinh Raj**: methodology, data curation, investigation, formal analysis, writing – review and editing. **Lucas Zhang**: investigation, writing – review and editing. **Sepideh Biabanialitappeh**: investigation, writing – review and editing. **J. David Bazak**: methodology, data curation, investigation, formal analysis, resources, writing – review and editing, software. **Nav Nidhi Rajput**: resources, methodology, investigation, formal analysis, supervision, funding acquisition, writing – review and editing, visualization. **Xiaoliang Wei**: conceptualization, methodology, data curation, investigation, validation, formal analysis, supervision, funding acquisition, visualization, project administration, resources, writing – original draft, writing – review and editing.

## Conflicts of Interest

The authors declare no conflicts of interest.

## Supporting information



The authors have cited additional references within the Supporting Information [19, 27, 30, 45, 52, 66, 70, 82, 83, 84, 85, 86, 87, 88, 89, 90, 91, 92, 93, 94].**Supporting File**: anie74935‐sup‐0001‐SuppMat.docx.

## Data Availability

The data that support the findings of this study are available from the corresponding author upon reasonable request.
