# Supplementary material for: Boosting Charge Storage in Ferri/Ferrocyanide‐Based Alkaline Redox Flow Batteries
Source: Angew Chem Int Ed Engl. 2026 Sep 22;65(45):e3588313. doi: 10.1002/anie.3588313 (PMC13725615; doi:10.1002/anie.3588313)
Supplement: Supplementary file 1 [file ANIE-65-e3588313-s001.docx]

**Electronic Supplementary Information for**

**Boosting Charge Storage in Ferri/Ferrocyanide-Based Alkaline Redox Flow Batteries**

Mahla Sarfaraz Khabbaz,^a§^ Kuldeepsinh Raj,^b§^ Lucas Zhang,^a^ Sepideh Biabanialitappeh,^a^ J. David Bazak,^c^ Nav Nidhi Rajput,^b^* Xiaoliang Wei^a^*

^a^ School of Mechanical Engineering, Purdue University, 723 West Michigan Street, Indianapolis, IN 46202

^b^ Department of Materials Science and Chemical Engineering, Stony Brook University, 450 Circle Road, Stony Brook, NY 11794

^c^ Physical and Computational Sciences Directorate, Pacific Northwest National Laboratory, 3340 Stevens Drive, Richland, WA 99354

^§^ These authors contributed equally to this work.

**Corresponding Authors:**

Dr. Nav Nidhi Rajput: [Navnidhi.Rajput@stonybrook.edu](mailto:Navnidhi.Rajput@stonybrook.edu)

Dr. Xiaoliang Wei: [wei304@purdue.edu](mailto:wei304@purdue.edu)

**Section S1. Experimental**

**S1.1 Materials and Methods**

*Materials*: Sodium ferrocyanide decahydrate (Na_4_Fe(CN)_6_·10H_2_O, ≥98.0%), sulfur (S, purified by sublimation, ~100 mesh particle size), and lithium sulfide (99.995%) were purchased from Sigma-Aldrich. Lithium chloride (LiCl, ≥99.0%), lithium hydroxide monohydrate (LiOH·H_2_O, ≥98%), sodium sulfide nonahydrate (Na_2_S·9H_2_O, 98+%), potassium ferricyanide (K_3_Fe(CN)_6_, 99+%), potassium ferrocyanide trihydrate (K_4_Fe(CN)_6_·3H_2_O, 99+%), lithium perchlorate anhydrous (LiClO_4_, 95.0%), cobalt (II) nitrate hexahydrate (Co(NO_3_)_2_·6H_2_O, 99%), and cobalt(II) chloride hexahydrate (CoCl_2_·6H_2_O, 98%) were purchased from Fisher Scientific. Fumasep^®^ F1850 cation exchange membrane with an ion exchange capacity (IEC) of 0.56 mmol·g^−1^ was obtained from Fuel Cell Store (Bryan, TX). Nafion^®^ 212 (NR212) membrane was purchased from Ion Power (New Castle, DE). AvCarb^®^ HCB 1186 carbon fabric electrode (1.32 mm thick) was purchased from Fuel Cell Earth (Woburn, MA).

*Methods*: ATR-FTIR spectra were collected using a Thermo Scientific Nicolet iS5 spectrometer equipped with an iD7-ATR accessory including a diamond crystal plate. Field emission scanning electron microscopy (FE-SEM) images and energy-dispersive X-ray spectroscopy (EDX) spectra were collected with a JEOL JSM-7800F spectrometer at 5.0 kV. X-ray diffraction (XRD) spectra were recorded using a Bruker D8 Discover X-ray diffractometer. The electrolyte pH was measured using an accumet^TM^ AE150 Benchtop pH Meter.

S1.2 Synthesis of Redox Materials

*Li_3_Fe(CN)_6_*: The synthetic procedures for Li_3/4_Fe(CN)_6_ were described in our previous report.^1^ Briefly, a solution of K_3_Fe(CN)_6_ (78.68 g, 0.24mol) in water (200mL) was mixed with a solution of LiClO_4_ (80.36 g, 0.75mol) in water (300mL) under an ice water bath for 30 min. The resulting white precipitate (KClO_4_) was removed by vacuum filtration and water was evaporated from the filtrate to give a solid. The solid was redissolved in cold water (90 mL) and the white solid was vacuum filtered. The re-dissolution and filtration process was repeated twice to ensure complete removal of KClO_4_. Final drying in a vacuum oven at 40 °C produced Li_3_Fe(CN)_6_ as a brown solid (50.2, 0.21 mol, 90%).

*Li_4_Fe(CN)_6_*: A solution of K_4_Fe(CN)_6_·3H_2_O (43.89g, 0.105mol) in water (200 mL) was combined with LiClO_4_ (44.99 g, 0.4mol) in water (175mL) under an ice water bath for 30 min. The resulting white precipitate (KClO_4_) was removed by vacuum filtration and water was evaporated from the filtrate to give a solid. The solid was redissolved in cold water (30 mL) and the white solid was vacuum filtered. The re-dissolution and filtration process was repeated twice to ensure complete removal of KClO_4_. Final drying in a vacuum oven at 40 °C produced Li_4_Fe(CN)_6_ as a teal-colored solid (22.7g, 0.13 mol, 90%).

*DHPS*: The synthetic procedure for DHPS was described in Ref. ^2^. 8.278 g of 2,5-dihydroxy-1,4-benzoquinone (0.059 mol) and 11.106 g of 3,4-diaminobenzenesulfonic acid (0.059 mol) were added to 260 mL of water in a 500 mL three-neck round-bottom flask under nitrogen. The reaction mixture was heated to 105 °C in an oil bath and refluxed overnight. After cooling to room temperature, the mixture was diluted with acetone. The product was washed three times with acetone under stirring and collected by vacuum filtration to obtain a dark-colored powder. Finally, the product was dried under vacuum in a vacuum oven at 40 °C overnight, yielding 13.325 g of DHPS (72.9% yield).

S1.3 Solubility and Viscosity Measurements

The solubilities of M_3/4_Fe(CN)_6_ and M_2_S_2/4_ in water and in 1 M MOH (M = Na, K, Li) were determined using ultraviolet–visible (UV–vis) spectroscopy on a Thermo Scientific Evolution 600 spectrometer. For each sample, the linear peak intensity–concentration calibration curve was pre-determined by collecting the UV-vis spectra of a series of millimolar-scale standard solutions. The saturated solution was diluted at a specified volumetric ratio (×1000-fold for most samples), and the resulting UV-vis peak intensity was interpolated into the calibration curve to determine the solubility.

The kinematic viscosities of alkaline electrolytes of Li_3_Fe(CN)_6_ in 1 M LiOH were measured using a Cannon 1C A901 Ubbelohde glass capillary viscometer. The viscometer was calibrated with deionized water with a viscometer constant of 0.03 cSt‧s^-1^. The dynamic viscosity was obtained by multiplying the kinematic viscosity and the density of this electrolyte.

S1.4 NMR Characterizations

NMR measurements were performed with a Bruker Avance NEO 11.7 T (500 MHz ^1^H) spectrometer (for ^1^H, ^7^Li, ^17^O) with a broadband tuning SmartProbe and a Bruker Avance NEO 14.1 T (600 MHz ^1^H) spectrometer (for ^13^C) with a TCI cryoprobe. Temperature was regulated to 298.2 K using the BCU/2 chiller system and the probe’s onboard heater. Pulsewidths for ^1^H were separately calibrated for all samples owing to the differential extent of radiation damping (π/2 ranging from 8.2 μs to 16 μs), whereas ^7^Li, ^13^C, and ^17^O were calibrated from standards (π/2 of 13.9 μs, 18.9 μs, and 31.7 μs, respectively), which also served as the reference markers (0.1 M LiCl (aq) at 0 ppm for ^7^Li; 1% CHCl_3_ in acetone-d6 at 79.19 ppm; and neat D_2_O at 0 ppm). For chemical shift determinations of the samples, a coaxial insert containing D_2_O for locking was used. Recycle delays in all cases were determined by relaxation measurements on each sample, using saturation recovery, and T_2_ relaxation was measured using the Carr-Purcell-Meiboom-Gill (CPMG) echo train method. Pulsed-field gradient (PFG) NMR diffusivity measurements were performed for ^1^H, ^7^Li, and ^13^C, with gradient durations of 2.0-2.4 ms for the former two and 2.4-3.2 ms for the latter. For ^7^Li and ^13^C, maximum gradient power of the solution probes (0.48 T/m) was used in all cases, and the diffusion time was scaled from 240 ms to 400 ms to attain suitable signal attenuation, whereas for the faster-diffusing ^1^H signals, a fixed diffusion time of 100 ms was employed with tailored maximum gradient strength (ranging from 44-98% of the maximum).

S1.5 MD Simulation

All classical MD simulations were carried out using LAMMPS, version 3 Mar2020,^3^ employing automated high-throughput workflows managed through the MISPR infrastructure.^4^ Starting configurations were constructed by randomly placing all molecular species inside a cubic simulation box of size 6 × 6 × 6 nm,^3^ with periodic boundary conditions applied in all three directions using the PACKMOL package.^5^ Force field parameters for Li^+^ and K^+^ were adopted from the work of Joung and Cheatham,^6^ and water molecules were represented using the SPC/Fw^7^ model. Lennard-Jones (LJ) parameters for ferrocyanide were taken from Huang et al.,^8^ while bonded interaction parameters were sourced from Prampolini et al.^9^ Parameters for OH^–^ were obtained from Bonthuis et al.^10^ LJ interactions were truncated at a cutoff distance of 1.2 nm, and long-range electrostatic interactions were computed with the particle-particle particle-mesh (PPPM) method.^11^ The prepared systems were undergoing a two-stage energy minimization: steepest-descent minimization to a force convergence criterion of 1000 kcal mol^-1^ Å^-1^, followed by conjugate-gradient minimization with a criterion of 10 kcal mol^-1^ Å^-1^. The minimized systems were then equilibrated at 298.15 K and 1 atm in the NPT ensemble for 2 ns using a time constant of 1 fs. The equilibrated systems were subsequently heated to 500.15 K for 2 ns and then quenched back to 298.15 K in four steps over 3 ns. Finally, production simulations were carried out in the NVT ensemble at 298.15 K for 10 ns, and the resulting trajectories were used to calculate the properties of interest.

Table S1. Number of molecules of each species used in the six MD-simulated electrolyte systems.

| **Electrolyte** | **Li^+^** | **K^+^** | **Fe(CN)_6_^4^⁻** | **H_2_O** | **OH⁻** |
| --- | --- | --- | --- | --- | --- |
| 3.37 M Li_4_Fe(CN)_6_ in H_2_O | 1752 | - | 438 | 2891 | - |
| 2.83 M Li_4_Fe(CN)_6_ in 1 M LiOH | 1602 | - | 368 | 3463 | 130 |
| 0.68 M K_4_Fe(CN)_6_ in H_2_O | - | 352 | 88 | 6224 | - |
| 0.36 M K_4_Fe(CN) _6_ in 1 M KOH | - | 318 | 47 | 6492 | 130 |
| 0.68 M Li_4_Fe(CN)_6_ in H_2_O | 352 | - | 88 | 6330 | - |
| 0.36 M Li_4_Fe(CN)_6_ in 1 M LiOH | 318 | - | 47 | 6621 | 130 |

The structural properties of the electrolytes were derived from MD trajectories and output files using MDPropTools version 0.0.4, an open-source Python package developed for MD analysis. Snapshots were sampled every 50 ps to analyze structural properties such as the RDF, CN and cluster analysis. The atom-pair radial distribution functions (RDFs) and coordination numbers (CNs) were computed according to the following expressions (Eqs. S1-2):

$\boldsymbol{g}_{\boldsymbol{ij}}\boldsymbol{=}\frac{\boldsymbol{V}}{\boldsymbol{NiNj}\boldsymbol{4}\boldsymbol{\pi}\boldsymbol{r}^{\boldsymbol{2}}\boldsymbol{d}} \sum_{\boldsymbol{i=1}}^{\boldsymbol{N}_{\boldsymbol{i}}} \sum_{\boldsymbol{j=1}}^{\boldsymbol{N}_{\boldsymbol{j}}} \boldsymbol{\delta}\left( \boldsymbol{r - rij} \right) \left\{ \boldsymbol{\delta=1, if}\boldsymbol{r}_{\boldsymbol{ij}}\boldsymbol{\leq r ;\delta=0, if}\boldsymbol{r}_{\boldsymbol{ij}}\boldsymbol{> r} \right\}$(S1)

$\boldsymbol{CN}_{\boldsymbol{ij}}\mathbf{=}\frac{\boldsymbol{N}_{\boldsymbol{j}}}{\boldsymbol{V}} \int_{\mathbf{0}}^{\boldsymbol{r}_{\boldsymbol{min}}} \boldsymbol{g}_{\boldsymbol{ij}}\left( \boldsymbol{r}^{\mathbf{'}} \right)\boldsymbol{d}\boldsymbol{r}^{\mathbf{'}}$	(S2)

where 𝑔_𝑖𝑗_ is the RDF between atom types 𝑖 and 𝑗, 𝑉 is the volume, 𝑁_𝑖_ and 𝑁_𝑗_ are the respective atom counts, 𝑑 is the histogram bin width, 𝑟 is the bin, 𝑟_𝑖𝑗_ is the interatomic distance and 𝐶𝑁_𝑖𝑗_ is the coordination number of species 𝑗 around 𝑖 at a cutoff distance 𝑟_𝑚𝑖𝑛_ corresponding to the position of the first minimum in 𝑔_𝑖𝑗_.

S1.5.1 Cluster analysis

First-shell cluster populations were obtained with the cluster_analysis module (<https://github.com/molmd/mdproptools/blob/master/mdproptools/structural/cluster_analysis.py>)of MDPropTools (v0.0.4), applied to the LAMMPS production trajectories through the MISPR analysis workflow. The procedure has two stages. (i) Cluster extraction. For each equilibrated MD trajectory, atomic clusters centered on the cation of interest (Li^+^ or K^+^) were identified using a coordination cutoff radius set at the position of the first minimum following the first solvation-shell peak in the corresponding radial distribution function (RDF) for each pair. Each resulting cluster was written as an .xyz file. (ii) Configuration assignment. The extracted clusters were passed to get_unique_configurations, which re-examines each cluster and counts, for every molecular species, how many molecules of that species donate a coordinating atom within cutoff radius of the central cation. Clusters sharing the same set of coordinating-molecule counts were grouped into a single unique configuration, and the frequency of every unique configuration was tabulated over the full set of sampled frames. The three most frequent configurations of each system are those reported in Table 1 of the main text and illustrated in Figure S6; and the OH⁻-containing entry in Table 1 is the summed frequency of all configurations containing at least one OH⁻ in the first shell.

S1.5.2 Speciation analysis (SSIP, CIP and AGG)

Ion-pair speciation was derived directly from the cluster configuration table described above, using a cation-centred criterion. For each unique first-shell configuration, n_anion denotes the number of Fe(CN)_6_^4^⁻ anions coordinating the central cation, and the configuration was assigned as a solvent-separated ion pair (SSIP) if n_anion = 0; as a contact ion pair (CIP) if n_anion = 1; and as an aggregate (AGG) if n_anion ≥ 2, that is, if the cation bridges two or more anions. The population of each speciation class was obtained as the sum of the frequencies of all configurations assigned to that class, so that SSIP + CIP + AGG = 100% by construction. These are the fractions plotted in Figure 2e.

S1.6 DFT Calculation

Binding energy calculations were performed using the binding energy workflow (<https://github.com/molmd/mispr/blob/master/mispr/gaussian/workflows/base/binding_energy.py> ) implemented in MISPR,^4^ with Gaussian 16 Revision C.01^12^ as the backend electronic-structure engine. All geometry optimizations and vibrational frequency calculations were performed at the B3LYP/6-311+G(d,p) level of theory and long-range dispersion interactions were accounted for using Grimme's D3 correction. Each pair was treated as a two-body complex consisting of one cation (Li^+^ or K^+^) and one partner species, either a single H_2_O molecule or a single Fe(CN)_6_^4^⁻ anion. The workflow proceeds as follows. (i) The input structures of each species were first optimized individually (ii) A vibrational frequency calculation at the same level confirms that each optimised molecular species is a true minimum with no imaginary frequencies (iii) The complex is assembled by placing the cation at a specified binding site of the partner, the oxygen atom of H_2_O, or a terminal nitrogen atom of Fe(CN)_6_^4^⁻ (iv) The complex is re-optimised and its vibrational frequencies verified at the same level with no imaginary frequencies. (v) The binding energy was then evaluated as, E_binding_ = E_complex_ - (E_partner(solvent/anion)_ + E_cation_), where E_complex_ is the energy of the molecular complex and E_partner(solvent/anion)_ and E_cation_ are the energies of the individually optimized solvent/anion molecule and cation, respectively.

All calculations were performed both in the gas phase and with implicit solvation using the SMD solvation model, with water as the solvent. The SMD-corrected binding energies are reported as the primary result in the main text (Figure S7, Table S2), as they account for the dielectric screening expected in the aqueous electrolyte and avoid the unscreened Coulombic artifacts that dominate gas-phase energies for a highly charged species such as Fe(CN)_6_^4^⁻. The corresponding gas-phase values are also tabulated in Table S2 for transparency and comparison.

Table S2. DFT binding energies at the B3LYP-D3/6-311+G(d,p) level, in the gas phase and in implicit water (SMD).

| **Complex** | **SMD-water**  **(KJ mol ^–1^)** | **Gas-phase**  **(KJ mol ^–1^)** |
| --- | --- | --- |
| Li^+^ - H_2_O | -67.0 | -153.7 |
| K^+^ - H_2_O | -37.4 | -81.0 |
| Li^+^ - Fe(CN)_6_^4^⁻ | -109.4 | -1579.6 |
| K^+^ - Fe(CN)_6_^4^⁻ | -68.4 | -1428.2 |

S1.7 CV Measurements

CV curves were measured with a three-electrode setup using a CHI760D potentiostat (CH Instruments, Austin, TX). The electrolyte consisted of 10 mM Li_3_Fe(CN)_6_, 5 mM DHPS, or 10 mM Li_2_S_2_ in either 1 M LiCl or 1 M LiOH. For Li_3_Fe(CN)_6_ and DHPS, a 3 mm diameter glassy carbon working electrode was used, while for Li_2_S_2_, a 2 mm diameter gold working electrode was used.

The redox kinetics of Li_3_Fe(CN)_6_ was determined using a combination of Randles–Ševčík and Nicholson analyses.^13^ CV curves were recorded at various scan rates (*ν*), and the diffusion coefficient (*D*) was calculated according to Eq. S3:

$i_{p}=2.69\times{10}^{5} AC({\nu D)}^{\frac{1}{2}}$	(S3)

Here, *i*_p_ represents the peak current density (in A), *A* is the GC area (0.07 cm^2^), *C* is the concentration (10 × 10^‒6^ mol‧cm^‒3^). *D* was calculated from the slope of the linear *i*_p_– *ν*^1/2^ plot. The electrochemical rate constant (*k*_0_) was determined using Eqs. S4 and S5:

$Ѱ=\frac{(-0.6288+0.0021\Delta E_{p})}{(1-0.017\Delta E_{p})}$	(S4)

$Ѱ={0.16k}_{0}{(D\nu)}^{-1/2}$	(S5)

where Ψ is the kinetic parameter that was calculated from the peak separation in CV (*ΔE*_p_). *k*_0_ was derived from the slope of the linear Ψ – *v*^–1/2^ plot.

S1.8 Flow Cells

The HCB 1186 electrodes used for the DHPS anolyte and Li_4_Fe(CN)_6_ catholyte were thermally treated in air at 400 °C for 6 h to enhance its wettability. To promote redox reactions, metallic cobalt electrocatalyst-decorated HCB 1186 (Co/HCB) was employed for polysulfide anolyte. To prepare the Co/HCB, HCB 1186 was treated in the following steps: boiling in 1 M NaOH for 1 h, rinsing with deionized water, soaking in a 1 M Co(NO₃)₂ solution for 24 h, drying in an oven at 80 °C for 24 h, and calcining in 4% H₂/argon atmosphere at 800 °C for 8 h. The resulting Co/HCB had a Co loading of 6.9 mg·cm⁻². The flow channel cavity for hosting the electrodes had a dimension of 3 cm × 2.2 cm × 1 mm (W × L × H), giving a compression ratio of ~25% and an active area of 6.6 cm^2^.

The alkaline S/Fe flow cells used 1 M Li_3_Fe(CN)_6_ catholyte (10 mL) and 1 M Li_2_S_2_ (15 mL), both in 1 M LiOH, which were separated by a Li^+^ form Fumasep^®^ F1850 membrane. The high-concentration S/Fe cell was started with 2.5 M Li_3_Fe(CN)_6_ catholyte (10 mL) and 2.5 M Li_2_S_2_ (15 mL), both in 1 M LiOH. The alkaline DHPS/Fe flow cells used 1 M Li_3_Fe(CN)_6_ catholyte (10 mL) and 1 M DHPS anolyte (10 mL) in 1 M LiOH with Li^+^ form Nafion^®^ 212 membrane. Electrolytes were circulated at a rate of 20 mL·min⁻¹ using a Masterflex^®^ L/S^®^ peristaltic pump (Cole-Parmer, Vernon Hills, IL). The flow cells were cycled at room temperature in a constant current mode with voltage cutoff controls using a Neware CT-4008 battery tester (Belleville, IL). The cutoff voltages were 0.5 – 1.4 V for the 1 M cell and 0.5 – 1.55 V for the 2.5 M cell.

Two-electrode electrochemical impedance spectroscopy (EIS) was employed to obtain Nyquist plots of the flow cells and calculate the area-specific resistance (ASR) using a Solartron SI 1287A electrochemical interface in conjunction with an SI 1250E frequency response analyzer (Scribner Associates, Southern Pines, NC). The frequency range was set to 0.1 – 6.55×10^4^ Hz. EIS was measured at the open-circuit potential of the flow cell with 10 mV alternate current (AC) perturbations.

The polarization curves at 100% SOC were obtained by recording the voltage profile under an applied linear current density ramp from 0 – 100 mA‧cm^-2^ at a rate of 1 mA‧cm^-2^‧s^-1^. The power density was obtained by multiplying the current density with the voltage.

S1.9 Symmetric Flow Cells

The symmetric Li_3/4_Fe(CN)_6_ flow cell was loaded with the same thermally treated HCB 1186 electrodes and the same electrolytes containing 7 mL of 1 M Li_3_Fe(CN)_6_, 1 M Li_4_Fe(CN)_6_, and 1 M LiOH at the two sides. A Li^+^ form Nafion 212 membrane of an active area of 6.6 cm^2^ was used. The electrolyte flow rate was 20 mL·min^-2^. A constant current of 20 mA·cm^-2^ with a voltage cutoff control was used to cycle the symmetric flow cells.

For symmetric polysulfide flow cells, 10 mL 1 M Li_2_S_2_ and 10 mL 0.5 M Li_2_S_4_, both in 1 M LiOH, were loaded at the two sides. The same Co/HCB electrodes were used at both sides, together with a Li^+^ form F1850 membrane having an active area of 6.6 cm^2^. The flow cells were tested at 20 mA·cm^-2^ inside and outside a Vigor^®^ N_2_-filled glovebox, respectively, to examine the effect of air exposure.

S1.10 Crossover Test

The crossover flux of Li_2_S_2_ through F1850 membrane under alkaline condition was measured using an H-shaped permeation cell.^14^ To avoid air oxidation of Li_2_S_2_, the permeation experiment was performed in a N₂-filled Vigor^®^ SG2400/750TS glove box (Houston, TX) maintaining an O₂ level below 1 ppm. The source chamber contained 15 mL of 1 M Li_2_S_2_ in 1 M LiOH, and the permeate chamber contained 15 mL of 2 M LiCl in 1 M LiOH to balance the osmotic pressure. A F1850 membrane with an effective diameter of 1 cm was placed between the two chambers. Prior to the measurements, the membranes were pretreated by boiling in 3 wt% hydrogen peroxide and then in 1 M H_2_SO_4_, both at 80 °C for 1 h, and finally were converted to Li⁺ form by immersion in 1 M LiOH for 24 h at room temperature. Both chambers were magnetically stirred.

At specified time intervals, 3 mL sample solutions were drawn from the permeate chamber, sealed in UV–vis cuvettes, and analyzed by UV–vis spectroscopy. After each measurement, the sample was returned to the permeate chamber to maintain a constant volume. The obtained UV-vis peak intensities were interpolated into the calibration plot (Section 2.3) to determine the permeation concentrations (*C*_t_). The permeability (*P*) was calculated using Eq. S6:^15^

$Ln\left( 1-\frac{C_{t}}{C_{0}} \right)=-\frac{AP}{TV}t$	(S6)

where *C*_0_ is the initial concentration in the source chamber (1 M), *A* is the effective membrane area (0.78 cm²), *T* is the membrane thickness (50 µm), and *V* is the electrolyte volume (15 mL). Plotting $-Ln\left( 1-\frac{C_{t}}{C_{0}} \right)$ versus time allowed *P* to be determined from the slope.

**Section S2. Supporting Figures**

**Figure S1.** UV-vis spectra of (a) Li_3_Fe(CN)_6_ and (b) Li_4_Fe(CN)_6_ at similar concentrations in neutral and 1 M LiOH electrolytes, respectively.

**Figure S2.** (a,c) The UV–vis spectra of standard solutions and (b,d) the derived calibration plots of Li_3_Fe(CN)₆ and Li_4_Fe(CN)₆, respectively, in 1 M LiOH. The red curves and red asterisks indicate the data collected from the samples diluted from saturation.

**Figure S3.** ATR-FTIR full spectrum of 0.4 M Li_4_Fe(CN)_6_ in 1 M LiOH.

**Figure S4.** ^1^H and ^13^C NMR of the Li_4_Fe(CN)_6_ in 1 M LiOH concentration series, echoing the trends exhibited by the ^7^Li and ^17^O concentration series spectra in Figure 2b,c.

**Figure S5.** RDFs showing cation-O(water), cation-N(CN^–^), and cation-O(OH^–^) peaks in neutral Li_4_Fe(CN)_6_ and K_4_Fe(CN)_6_ electrolytes.

**Figure S6.** Representative top three cluster structures for each electrolyte system, showing the most probable local solvation environments around the cation.

**Figure S7.** DFT-computed binding energies of M⁺ (Li^+^ and K^+^) with H_2_O and Fe(CN)_6_^4–^ in implicit water (SMD solvation model) at the B3LYP-D3/6-311+G(d,p) level.

**Figure S8.** Randles-Sevcik and Nicholson analyses for 10 mM Li_3_Fe(CN)_6_ in 1 M LiOH: (a) CV curves at different scan rates; (b) the peak current density *i*_p_ vs. *v*^1/2^; (c) the kinetic parameter *Ψ* vs. *ΔE*_p_; and (d) *Ψ* vs. *v^-1/2^*.

**Figure S9.** Randles-Sevcik and Nicholson analyses for 10 mM Li_3_Fe(CN)_6_ in 1 M LiCl: (a) CV curves at different scan rates; (b) the peak current density *i*_p_ vs. *v*^1/2^; (c) the kinetic parameter *Ψ* vs. *ΔE*_p_; and (d) *Ψ* vs. *v^-1/2^*.

**Figure S10.** (a,c) The UV–vis spectra of standard solutions and (b,d) the derived calibration plots of Li_2_S_2_ and Li_2_S_4_, respectively, in 1 M LiOH. Multiple polysulfide species are detected in a similar pattern in nominal Li_2_S_2_ and Li_2_S_4_ alkaline electrolyte: the 300 nm peak assigned to S_2_^2–^ and the 368 nm peak to S_4_^2–^.^16, 17^ The 368 nm peak was used to measure the solubilities. The red curves and red asterisks indicate the data collected from the samples diluted from saturation. (e) Summarized solubilities of M_2_S_n_ (M = Li and Na; n = 1, 2, and 4) in water and in 1 M MOH.

**Figure S11.** (a) FESEM image of pristine AvCarb HCB 1186 electrode and (b) an expanded view.

**Figure S12.** Voltage curves at different current densities for a 1 M S/Fe flow cell in 1 M LiOH.

**Figure S13.** (a) Cycling capacities and efficiencies and (b) voltage curves of selected cycles of 1 M neutral S/Fe flow cell. The current density was 20 mA‧cm^–2^.

**Figure S14.** Voltage curves of selected cycles along the cycling of S/Fe flow cells in 1 M LiOH: (a) 1 M and (b) 2.5 M.

**Figure S15**. Rate capability of 2 M alkaline S/Fe flow cell.

**Figure S16.** The voltage curves of 2 M alkaline S/Fe flow cell at 20 mA‧cm^-2^ under different flow rates.

**Figure S17.** Alkaline symmetric 2 M Li_3/4_Fe(CN)_6_ flow cells: (a) EIS showing an ASR of 4.3 Ω‧cm^2^; (b) Voltage curves along the cycling.

**Figure S18.** (a) FESEM image, (b) EDX C mapping, (c) EDX S mapping, and (d) EDX sum spectrum of the anolyte-side HCB electrode after cycling a 1 M neutral S/Fe flow cell. C and S are the only major elements detected. Surprisingly, cobalt electrocatalyst was not detected on the cycled electrode (the reason was not well understood).

**Figure S19.** FESEM images of the anolyte-side Sigracell GFD graphite felt electrode after cycling a neutral S/Fe flow cell. The catholyte was 8 mL of 2 M Li_3_Fe(CN)_6_ and the anolyte was 16 mL of 2 M Li_2_S_2_. The membrane was F1850. The current density was 20 mA‧cm^-2^. The cycling performance can be found in Ref. ^1^.

**Figure S20.** (c) Voltage curves of symmetric Li_2_S_2/4_ flow cells in air and N_2_ atmospheres: (a) In neutral electrolyte; (b) In alkaline electrolyte.

**Figure S21.** Li_2_S_2_ crossover tests: (a) UV-vis spectra of Li_2_S_2_ standard solutions, (b) intensity-concentration calibration plot using the 300 nm peak, and (c) time-dependent UV-vis spectra of permeated Li_2_S_2_ in an alkaline electrolyte containing 2 M LiCl and 1 M LiOH. (d-f) Same with a neutral electrolyte containing 2 M LiCl.

**Figure S22.** A photograph of cell parts after cycling a 1 M alkaline S/Fe flow cell (shown in Figure 3d), showing a considerable amount of yellow sulfur deposits at catholyte side.

**Figure S23.** (a) FESEM image, (b) EDX C mapping, (c) EDX S mapping, and (d) EDX sum spectrum of the catholyte-side HCB electrode after cycling a 1 M neutral S/Fe flow cell. C and S are the only major elements detected.

**Section S3. Redox Materials-Based Cost Analysis**

The price of Li_2_S ($21.7 kg^-1^) was adopted from a recent review article.^18^ Other literature reports predicted even lower prices for Li_2_S, e.g., as low as $6.6 kg^‒1^,^19^ potentially offering substantial space for further cost reduction. The prices of synthetic Li_3/4_Fe(CN)_6_ were directly adopted from a prior report.^20^ The price of LiOH‧H_2_O ($16.9 kg^-1^) was taken from Scap Monster, averaged over the past year.^21^ The redox materials cost is calculated as the total cost of disulfide and ferricyanide needed for achieving per unit of stored energy ($ kWh^‒1^). Based on the voltage curves of our S/Fe flow cells, an average cell voltage of 0.95 V is used to calculate the energy. For the alkaline system, we used 2.5 M Li_3_Fe(CN)_6_ in 1 M LiOH as an example electrolyte, giving rise to a Li_3_Fe(CN)_6_-to-LiOH molar ratio of 2.5. The results are summarized in Table S3.

**Table S3.** Redox materials-based cost estimation for neutral and alkaline S/Fe RFBs.

| **Redox Materials** | | **Neutral S/Fe** | **Alkaline S/Fe** |
| --- | --- | --- | --- |
| **Li_2_S** | Mol. Wt. | 46 | 46 |
|  | Weight (kg‧kAh^‒1^) | 0.86 | 0.86 |
|  | Price ($ kg^‒1^) | 21.7 | 21.7 |
|  | Cost ($ kAh^‒1^) | 18.7 | 18.7 |
| **Sulfur** | Mol. Wt. | 32 | 32 |
|  | Weight (kg‧kAh^‒1^) | 0.60 | 0.6 |
|  | Price ($ kg^‒1^) | 0.2 | 0.2 |
|  | Cost ($ kAh^‒1^) | 0.12 | 0.12 |
| **Li_3_Fe(CN)_6_** | Mol. Wt. | 233 | 233 |
|  | Weight (kg‧kAh^‒1^) | 8.7 | 8.7 |
|  | Price ($ kg^‒1^) | 1.41^2^ | 1.41^2^ |
|  | Cost ($ kAh^‒1^) | 12.2 | 12.2 |
| **LiOH‧H_2_O** | Mol. Wt. | - | 42 |
|  | Weight (kg‧kAh^‒1^) | - | 0.63 |
|  | Price ($ kg^‒1^) | - | 16.9 |
|  | Cost ($ kAh^‒1^) | - | 10.6 |
| **Total Cost ($ kAh^‒1^)** | | 31.0 | 41.6 |
| **Total Cost ($ kWh^‒1^)** | | 32.7 | 43.8 (**This work**) |

**Section S4. Optimized Cartesian coordinates** **(coordinates are given in Å)**

**S4.1 Implicit water (SMD)**

H_2_O

| **Atom** | **X (Å)** | **Y (Å)** | **Z (Å)** |
| --- | --- | --- | --- |
| H | -1.714750 | 1.145192 | -0.003985 |
| O | -2.679005 | 1.181522 | 0.020239 |
| H | -2.959305 | 0.412956 | -0.492214 |

Li^+^ - H_2_O complex

| **Atom** | **X (Å)** | **Y (Å)** | **Z (Å)** |
| --- | --- | --- | --- |
| H | -1.742183 | 1.176812 | 0.052209 |
| O | -2.702289 | 1.250720 | -0.021221 |
| H | -3.005418 | 0.435866 | -0.441775 |
| Li | -3.765620 | 2.450502 | 0.883678 |

K^+^

| **Atom** | **X (Å)** | **Y (Å)** | **Z (Å)** |
| --- | --- | --- | --- |
| K | 1.068470 | 0.021630 | -0.069600 |

Li^+^

| **Atom** | **X (Å)** | **Y (Å)** | **Z (Å)** |
| --- | --- | --- | --- |
| Li | 1.045310 | 0.025190 | -0.085510 |

Fe(CN)_6_^4-^

| **Atom** | **X (Å)** | **Y (Å)** | **Z (Å)** |
| --- | --- | --- | --- |
| Fe | 0.799154 | 0.272000 | 0.064000 |
| C | 0.809910 | -1.314112 | -1.073764 |
| C | 0.799571 | -0.865789 | 1.650114 |
| C | -1.152672 | 0.263500 | 0.058442 |
| C | 0.788397 | 1.858112 | 1.201764 |
| C | 0.798737 | 1.409788 | -1.522114 |
| C | 2.750980 | 0.280500 | 0.069558 |
| N | 0.816679 | -2.269238 | -1.758533 |
| N | 0.799805 | -1.550598 | 2.605236 |
| N | -2.327989 | 0.258399 | 0.055126 |
| N | 0.781629 | 2.813238 | 1.886533 |
| N | 0.798502 | 2.094598 | -2.477236 |
| N | 3.926297 | 0.285601 | 0.072874 |

K^+^ - H_2_O complex

| **Atom** | **X (Å)** | **Y (Å)** | **Z (Å)** |
| --- | --- | --- | --- |
| H | -1.741730 | 1.171913 | 0.035203 |
| O | -2.705097 | 1.235729 | 0.006687 |
| H | -2.993182 | 0.444227 | -0.466316 |
| K | -4.208469 | 2.971560 | 1.237052 |

K^+^ - Fe(CN)_6_^4-^ complex

| **Atom** | **X (Å)** | **Y (Å)** | **Z (Å)** |
| --- | --- | --- | --- |
| Fe | 0.802627 | 0.276295 | 0.097675 |
| C | 0.804092 | -1.274348 | -1.091739 |
| C | 0.802825 | -0.910979 | 1.647140 |
| C | -1.149405 | 0.274992 | 0.097093 |
| C | 0.800280 | 1.815671 | 1.267271 |
| C | 0.802381 | 1.463831 | -1.451172 |
| C | 2.754657 | 0.277597 | 0.098540 |
| N | 0.804920 | -2.203877 | -1.810197 |
| N | 0.802876 | -1.626827 | 2.578824 |
| N | -2.324371 | 0.277275 | 0.098837 |
| N | 0.797807 | 2.753887 | 1.972342 |
| N | 0.802175 | 2.185202 | -2.378577 |
| N | 3.929619 | 0.281245 | 0.100990 |
| K | 0.724176 | 5.075200 | 3.152451 |

Li^+^ - Fe(CN)_6_^4-^ complex

| **Atom** | **X (Å)** | **Y (Å)** | **Z (Å)** |
| --- | --- | --- | --- |
| Fe | 0.799042 | 0.288304 | 0.086006 |
| C | 0.809723 | -1.291804 | -1.068060 |
| C | 0.799495 | -0.863078 | 1.662726 |
| C | -1.152756 | 0.276537 | 0.079659 |
| C | 0.788673 | 1.842271 | 1.204435 |
| C | 0.798481 | 1.436299 | -1.492265 |
| C | 2.750846 | 0.293621 | 0.090699 |
| N | 0.816409 | -2.237925 | -1.763632 |
| N | 0.799687 | -1.554269 | 2.612321 |
| N | -2.327313 | 0.272787 | 0.078810 |
| N | 0.781913 | 2.802046 | 1.880884 |
| N | 0.798129 | 2.134512 | -2.436693 |
| N | 3.925377 | 0.300225 | 0.096558 |
| Li | 0.771155 | 4.356184 | 2.910938 |

**S4.2 Gas phase**

H_2_O

| **Atom** | **X (Å)** | **Y (Å)** | **Z (Å)** |
| --- | --- | --- | --- |
| H | -1.714904 | 1.147534 | -0.002423 |
| O | -2.676263 | 1.178248 | 0.018056 |
| H | -2.961893 | 0.413888 | -0.491592 |

Li^+^ - H_2_O complex

| **Atom** | **X (Å)** | **Y (Å)** | **Z (Å)** |
| --- | --- | --- | --- |
| H | -1.738636 | 1.186992 | 0.023886 |
| O | -2.705498 | 1.212648 | 0.040992 |
| H | -2.998697 | 0.445656 | -0.470410 |
| Li | -3.770079 | 2.465249 | 0.876183 |

K^+^

| **Atom** | **X (Å)** | **Y (Å)** | **Z (Å)** |
| --- | --- | --- | --- |
| K | 1.068470 | 0.021630 | -0.069600 |

Li^+^

| **Atom** | **X (Å)** | **Y (Å)** | **Z (Å)** |
| --- | --- | --- | --- |
| Li | 1.045310 | 0.025190 | -0.085510 |

K^+^ - Fe(CN)_6_^4-^ complex

| **Atom** | **X (Å)** | **Y (Å)** | **Z (Å)** |
| --- | --- | --- | --- |
| Fe | 0.653919 | 0.303435 | 0.285855 |
| C | 1.033221 | -0.993424 | -1.142619 |
| C | 0.698525 | -1.113003 | 1.648928 |
| C | -1.279565 | 0.034122 | 0.042517 |
| C | 0.326508 | 1.663086 | 1.702045 |
| C | 0.656073 | 1.780912 | -1.047756 |
| C | 2.585509 | 0.711862 | 0.534916 |
| N | 1.282854 | -1.750625 | -1.997627 |
| N | 0.746950 | -1.942055 | 2.471751 |
| N | -2.432732 | -0.092863 | -0.101198 |
| N | 0.277274 | 2.589836 | 2.418469 |
| N | 0.767807 | 2.765222 | -1.674815 |
| N | 3.659713 | 1.157585 | 0.682794 |
| K | 2.209806 | 3.577653 | 0.612659 |

Li^+^ - Fe(CN)_6_^4-^ complex

| **Atom** | **X (Å)** | **Y (Å)** | **Z (Å)** |
| --- | --- | --- | --- |
| Fe | 0.974291 | 0.366396 | 0.135267 |
| C | 0.587725 | -1.218148 | -0.981292 |
| C | 0.966183 | -0.759041 | 1.768367 |
| C | -0.934023 | 0.757066 | 0.435385 |
| C | 1.226758 | 1.969402 | 1.253742 |
| C | 0.949588 | 1.531375 | -1.469700 |
| C | 2.917460 | 0.076776 | -0.081810 |
| N | 0.323839 | -2.150048 | -1.637523 |
| N | 0.925768 | -1.377012 | 2.761576 |
| N | -1.960352 | 1.227639 | 0.774430 |
| N | 1.069940 | 2.923419 | 1.928278 |
| N | 0.897507 | 2.260770 | -2.383623 |
| N | 4.073622 | -0.065638 | -0.190066 |
| Li | -0.832158 | 2.541533 | 1.682724 |

Fe(CN)_6_^4-^

| **Atom** | **X (Å)** | **Y (Å)** | **Z (Å)** |
| --- | --- | --- | --- |
| Fe | 0.799154 | 0.272000 | 0.064000 |
| C | 0.800880 | -1.354005 | -1.113404 |
| C | 0.790466 | -0.890681 | 1.700542 |
| C | -1.208252 | 0.250298 | 0.060146 |
| C | 0.797428 | 1.898005 | 1.241404 |
| C | 0.807841 | 1.434681 | -1.572542 |
| C | 2.806559 | 0.293703 | 0.067854 |
| N | 0.800362 | -2.304175 | -1.803307 |
| N | 0.783841 | -1.569506 | 2.658636 |
| N | -2.382376 | 0.235463 | 0.058243 |
| N | 0.797946 | 2.848175 | 1.931307 |
| N | 0.814467 | 2.113505 | -2.530636 |
| N | 3.980683 | 0.308537 | 0.069757 |

K^+^ - H_2_O complex

| **Atom** | **X (Å)** | **Y (Å)** | **Z (Å)** |
| --- | --- | --- | --- |
| H | -1.735131 | 1.171871 | 0.013770 |
| O | -2.699741 | 1.205839 | 0.036536 |
| H | -2.982719 | 0.437883 | -0.475627 |
| K | -4.228338 | 3.004449 | 1.235688 |

**References**

(1) Khabbaz, M. S.; Yue, D.; Shukla, L.; Thakur, A.; Biabanialitappeh, S.; Xie, J.; Bazak, J. D.; Wei, X. An energy-dense polysulfide/ferricyanide redox flow battery enabled by cation engineering. *Nano Energy* **2026**, *149*, 111722. DOI: <https://doi.org/10.1016/j.nanoen.2026.111722>.

(2) Hollas, A.; Wei, X. L.; Murugesan, V.; Nie, Z. M.; Li, B.; Reed, D.; Liu, J.; Sprenkle, V.; Wang, W. A biomimetic high-capacity phenazine-based anolyte for aqueous organic redox flow batteries. *Nature Energy* **2018**, *3* (6), 508-514. DOI: 10.1038/s41560-018-0167-3.

(3) Thompson, A. P.; Aktulga, H. M.; Berger, R.; Bolintineanu, D. S.; Brown, W. M.; Crozier, P. S.; Veld, P. J. I.; Kohlmeyer, A.; Moore, S. G.; Nguyen, T. D.; et al. LAMMPS-a flexible simulation tool for particle-based materials modeling at the atomic, meso, and continuum scales. *Computer Physics Communications* **2022**, *271*. DOI: 10.1016/j.cpc.2021.108171.

(4) Atwi, R.; Bliss, M.; Makeev, M.; Rajput, N. N. MISPR: an open-source package for high-throughput multiscale molecular simulations. *Scientific Reports* **2022**, *12* (1). DOI: 10.1038/s41598-022-20009-w.

(5) Martínez, L.; Andrade, R.; Birgin, E. G.; Martínez, J. M. PACKMOL: A Package for Building Initial Configurations for Molecular Dynamics Simulations. *Journal of Computational Chemistry* **2009**, *30* (13), 2157-2164. DOI: 10.1002/jcc.21224.

(6) Joung, I. S.; Cheatham, T. E. Determination of alkali and halide monovalent ion parameters for use in explicitly solvated biomolecular simulations. *Journal of Physical Chemistry B* **2008**, *112* (30), 9020-9041. DOI: 10.1021/jp8001614.

(7) Wu, Y. J.; Tepper, H. L.; Voth, G. A. Flexible simple point-charge water model with improved liquid-state properties - art. no. 024503. *Journal of Chemical Physics* **2006**, *124* (2). DOI: 10.1063/1.2136877.

(8) Huang, B. T.; Myint, K. H.; Wang, Y. M.; Zhang, Y. R.; Rao, R. R.; Sun, J.; Muy, S.; Katayama, Y.; Garcia, J. C.; Fraggedakis, D.; et al. Cation-Dependent Interfacial Structures and Kinetics for Outer-Sphere Electron-Transfer Reactions. *Journal of Physical Chemistry C* **2021**, *125* (8), 4397-4411. DOI: 10.1021/acs.jpcc.0c10492.

(9) Prampolini, G.; Yu, P. Y.; Pizzanelli, S.; Cacelli, I.; Yang, F.; Zhao, J. A.; Wang, J. P. Structure and Dynamics of Ferrocyanide and Ferricyanide Anions in Water and Heavy Water: An Insight by MD Simulations and 2D IR Spectroscopy. *Journal of Physical Chemistry B* **2014**, *118* (51), 14899-14912. DOI: 10.1021/jp511391b.

(10) Bonthuis, D. J.; Mamatkulov, S. I.; Netz, R. R. Optimization of classical nonpolarizable force fields for OH- and H3O+. *Journal of Chemical Physics* **2016**, *144* (10). DOI: 10.1063/1.4942771.

(11) Buneman, O. COMPUTER-SIMULATION USING PARTICLES - HOCKNEY,RW, EASTWOOD,JW. *Siam Review* **1983**, *25* (3), 425-426.

(12) M. J. Frisch, G. W. T., H. B. Schlegel, G. E. Scuseria, M. A.; Robb, J. R. C., G. Scalmani, V. Barone, G. A. Petersson, H.; Nakatsuji, X. L., M. Caricato, A. V. Marenich, J. Bloino, B. G. Janesko,; R. Gomperts, B. M., H. P. Hratchian, J. V. Ortiz, A. F. Izmaylov,; J. L. Sonnenberg, W., F. Ding, F. Lipparini, F. Egidi, J. Goings,; B. Peng, A. P., T. Henderson, D. Ranasinghe, et al. Gaussian 16 Revision. C.01, Gaussian, Inc, Wallingford, CT 2016.

(13) Wang, H.; Sayed, S. Y.; Luber, E. J.; Olsen, B. C.; Shirurkar, S. M.; Venkatakrishnan, S.; Tefashe, U. M.; Farquhar, A. K.; Smotkin, E. S.; McCreery, R. L.; et al. Redox Flow Batteries: How to Determine Electrochemical Kinetic Parameters. *Acs Nano* **2020**, *14* (3), 2575-2584. DOI: 10.1021/acsnano.0c01281.

(14) Sarfaraz Khabbaz, M.; Thakur, A.; Maity, D.; Biabanialitappeh, S.; Mortha, B.; McKay, J. P.; Parada, G.; Agarwal, M.; Zhang, H.; Zhang, X.; et al. A polysulfide/ferricyanide redox flow battery with extended cycling. *J Mater Chem A* **2025**, *13*, 29610-29620, 10.1039/D5TA05404D. DOI: 10.1039/D5TA05404D.

(15) Li, Z. J.; Lu, Y. C. Polysulfide-based redox flow batteries with long life and low levelized cost enabled by charge-reinforced ion-selective membranes. *Nature Energy* **2021**, *6* (5), 517-528. DOI: 10.1038/s41560-021-00804-x.

(16) Steudel, R. Inorganic polysulfides S n 2− and radical anions S n·−. *Elemental Sulfur und Sulfur-Rich Compounds II* **2003**, 127-152.

(17) Kamyshny, A.; Goifman, A.; Gun, J.; Rizkov, D.; Lev, O. Equilibrium distribution of polysulfide ions in aqueous solutions at 25 C: a new approach for the study of polysulfides' equilibria. *Environ Sci Technol* **2004**, *38* (24), 6633-6644.

(18) Weng, W.; Lan, Y.; Tang, D.; Fu, L.; Tan, W.; Zhong, S. Research Progress on Lithium Sulfide Synthesis: A Review. *ACS Appl Energ Mater* **2025**, *8* (18), 13139-13154. DOI: 10.1021/acsaem.5c02015.

(19) Yuan, K.; Yuan, L.; Chen, J.; Xiang, J.; Liao, Y.; Li, Z.; Huang, Y. Methods and Cost Estimation for the Synthesis of Nanosized Lithium Sulfide. *Small Structures* **2021**, *2* (3), 2000059. DOI: <https://doi.org/10.1002/sstr.202000059> (acccessed 2025/11/26).

(20) Li, X. T.; Yao, Y.; Liu, C. X.; Jia, X.; Jian, J. H.; Guo, B.; Lu, S. T.; Qin, W.; Wang, Q.; Wu, X. H. Lithium Ferrocyanide Catholyte for High-Energy and Low-cost Aqueous Redox Flow Batteries. *Angew Chem Int Edit* **2023**, *62* (25). DOI: 10.1002/anie.202304667.

(21) Lithium hydroxides monohydrates LiOH 56.5% min, Magnets 0.0001% max Prices. Scap Monster, accessed on August 11, 2026.
